# An evaluation of programmatic assessment across health professions education using contribution analysis

**DOI:** 10.1007/s10459-025-10444-5

**Published:** 2025-06-04

**Authors:** Janica Jamieson, Claire Palermo, Margaret Hay, Rachel Bacon, Janna Lutze, Simone Gibson

**Affiliations:** 1 School of Medical and Health Sciences, Edith Cowan University, 270 Joondalup Drive, Joondalup, Perth, WA 6027 Australia; 2 Faculty of Medicine, Nursing and Health Sciences, Monash University, Melbourne, Australia; 3 Faculty of Health, University of Canberra, Canberra, Australia; 4 Faculty of Science, Medicine and Health, University of Wollongong, Wollongong, Australia; 5 Faculty of Medicine, Nursing and Health Sciences, Monash University, Monash, Australia

**Keywords:** Health professions education, Competency-based education, Programmatic assessment, Contribution analysis, Evaluation, Theory of change

## Abstract

Programmatic assessment is gaining traction in health professions education. Despite this popularity, educators continue to grapple with complex contextual factors that impact implementation and outcome attainment. We used contribution analysis, a theory-informed evaluation method, to understand the mechanisms underpinning successful implementation. Applying the six steps of contribution analysis, we developed a postulated theory of change (ToC) and then conducted a qualitative study with programmatic assessment stakeholders (graduates n = 15, supervisors n = 32, faculty n = 19) from four Australian dietetic programs. These data were analysed using the Framework Analysis method and integrated with data derived from a literature review across health disciplines, to assemble contribution claims and the story, and verify the ToC. Impact pathways for programmatic assessment from inception to implementation, and contribution to outcomes were articulated in the ToC. Leaders drove implementation using compromise and worked with a design team to apply the versatile principles. All people required training, and purposefully designed tools were implemented within an ideologically aligned system. Re-orientation of responsibilities situated learners as leaders, contributing to a psychologically safe environment which promoted growth mindsets. Credible high-stakes progression decisions were enabled, people experienced less stress, and derived gratification from assessment. External factors (institutional and accreditation requirements) and threats (resource mismatches, ideological misalignments, and capabilities of the people) were identified. Contribution analysis revealed mechanisms that educators can apply to implement a contextually responsive programmatic assessment across diverse settings.

**Supplementary Information:**

The online version contains supplementary material available at 10.1007/s10459-025-10444-5.

## Introduction

Competency-based education (CBE) has transformed the health professions education (HPE) landscape as educators explore and apply new approaches to teach and assess learners (Touchie & Ten Cate, 2016; Van Melle et al., 2019), notably exemplified by the advent of programmatic assessment (van der Vleuten & Schuwirth, 2005). Programmatic assessment is a systems approach to assessment which longitudinally employs fit-for-purpose assessment methods and moments, undertaken by equipped people, to drive and capture learning (van der Vleuten et al., 2012). Assessment moments are designed to provide rich feedback and are optimised for learning to illuminate learner development. Through a process of triangulation, assessments inform progression decisions which are proportional to the significance of the outcomes (Heeneman et al., 2021; van der Vleuten et al., 2012). Programmatic assessment is a promising remedy to the challenges encountered in operationalising competency-based assessment (Iobst & Holmboe, 2020). Emerging from theory (van der Vleuten & Schuwirth, 2005), the literature now widely describes the principles underpinning programmatic assessment (Heeneman et al., 2021). The recent proliferation within medicine, veterinary science, dietetics, paramedicine, dentistry, teaching and communication science (Baartman et al., 2022; Torre et al., 2021) demonstrates diffusion of theory into practice. Programmatic assessment is the leading approach to competency-based assessment (Caretta-Weyer et al., 2024; Pearce & Tavares, 2021), and the growing adoption highlights the need to understand the complex system in which it operates.

Research substantiates the feedback and learning function of programmatic assessment (Baartman et al., 2023; Schut et al., 2021), demonstrating that robust progression decisions are possible with sufficient quality data (Baartman et al., 2023; de Jong et al., 2022; Schut et al., 2021). Studies affirm the capacity for early detection and supportive remediation of underperformance (Schut et al., 2021). Yet early adopters have encountered challenges such as stakeholder resistance (Baartman et al., 2023; Jamieson et al., 2022; Schut et al., 2021) and design choice dilemmas when applying theoretical principles to authentic practice (Baartman et al., 2022; Schut et al., 2021). The continued perception or use of summative assessments has proven difficult to shake and undermines assessment’s intended learning function (Baartman et al., 2023; Bate et al., 2020; Schut et al., 2021). There is propensity for over-assessment which can burden all stakeholders (Bate et al., 2020; Schut et al., 2021). These implementation challenges may impede widespread adoption and hinder attainment of desired outcomes (Ryan et al., 2023). Such challenges arise as programmatic assessment is situated within (often established) cultures and is reliant on people for operationalisation (Jamieson et al., 2022; Torre et al., 2021). External factors become entwined, exerting unrecognised influence. The nexus between these influences - principles, cultures, people, external factors - coalesce unpredictably to make programmatic assessment non-standardised, context-specific, and inherently complex (Baartman et al., 2022; Schuwirth Torre et al., 2020). This complexity makes evaluation challenging and yet, as popularity grows, there is a need to understand the mechanisms upon which successful implementation is contingent (Schuwirth Torre et al., 2020; Torre et al., 2021; Van Melle et al., 2021). Without this understanding, we will be constrained in our ability to implement programmatic assessment and for it to fulfil its promise for competency-based education (Sandars, 2018).

### Evaluation in health professions education

Health professions education has historically applied reductionist evaluation approaches which presuppose a linear attribution relationship between actions and outcomes, relying on the control of variables and use of comparator groups (Frye & Hemmer, 2012; Van Melle et al., 2021). These approaches are incompatible with the complexity of HPE where contextual factors dynamically interact and exert variable influence on outcomes (Frye & Hemmer, 2012; Hall et al., 2021; Van Melle et al., 2021). Theory-informed evaluations are posited to accommodate inherent HPE complexity and have been increasingly applied within HPE scholarship over the last two decades, including contribution analysis, CIPP (context, input, process, product), layered analysis, RE-AIM (reach, effectiveness, adoption, implementation, maintenance) framework, and realist evaluation, to name a few (Allen et al., 2022; Frye & Hemmer, 2012; Haji et al., 2013). As opposed to ‘black box’ evaluation methods, these approaches intentionally explore and reveal the covert mechanisms between program activities and outcomes, making them well suited to understanding and evaluating complex programs (Frye & Hemmer, 2012). Broad features of theory-informed evaluation approaches include stepwise methods, stakeholder engagement, and development of program theory underpinned by data derived from diverse sources. Differences are observed in the foundational concepts and central evaluation questions. Table 1 presents a comparison of theory-informed evaluation methods, selected for their varying application within HPE scholarship to illustrate similarities and differences.

**Table 1:** Comparison of three theory-informed evaluation approaches used within health professions education: Contribution analysis, realist evaluation, and CIPP (context, input, process, and product)

Evaluation approach	Contribution analysis (Mayne, 1999)	Realist evaluation (Pawson & Tilley, 1997)	CIPP (Stufflebeam, 2014)
Origins	Developed in the 1990s to provide outcome evidence for complex programs, specifically monitoring government programs.	Developed in the 1990s in response to limitations of traditional evaluation approaches.	Developed in the 1960s in response to limitations of experimental evaluation approaches, specifically for national program evaluation.
Grand theory	Not defined; critical realism (subjectivity) suggested (Brousselle & Buregeya, 2018).	Scientific or critical realism (objectivity or subjectivity) (Pawson, 2006; Rees et al., 2023).	Objectivism (Stufflebeam, 2014).
Premise	Multiple factors influence outcomes for complex programs making attribution unachievable, instead determine program contribution to observed outcomes.	Programs work differently for different people and context.	Evaluation should not only measure program outcomes but also be used for continuous program improvement.
Intent	Determine the contribution an evaluand makes to observed outcomes.	Understand relationship between mechanisms and context on evaluand outcomes (CMO configuration).	Evaluation of evaluand context, inputs, processes, and products.
Central question	How and why has the program (or component) made a difference, or not, and for whom?	What works for whom, in what circumstances and why?	Dependent on prospective or retrospective application: What needs to be done? How should it be done? Is it being done? Is it succeeding?
Use of theory	Theory of Change (ToC) is developed which sequentially maps impact pathways from activities to stratified outcomes.	Program theory is developed which frames underlying logic from activities/ inputs to outcomes.	Program theory is developed which frames underlying logic from activities/ inputs to outcomes.
Causality	Inferred by generating credible and plausible claims about how evaluand contributes to observed outcomes.	Identification of mechanisms and influence of context on outcomes.	Determine relationships between evaluation (CIPP) components.
Methodology	Structured six step framework which develops and verifies ToC for evaluand with resultant contribution claims and contribution story.	Broad application of four steps: program theory formation, CMO hypothesis development, observation, and programme specification.	Prospective or retrospective application of evaluation studies for context, input, process, and product.
Methods	Not defined; responsive to evaluand.	Not defined; responsive to evaluand.	Not defined; responsive to evaluand.
Data type	Mixed, as suitable.	Mixed, as suitable.	Mixed, as suitable.
Stakeholder engagement	Yes	Yes	Yes
Iterative	Yes	Yes	Yes
Recognition of complexity	Yes	Yes	Yes
Application in HPE	Limited (Choi et al., 2023).	Increasing (Ajjawi & Kent, 2022).	Wide (Toosi et al., 2021).

Abbreviations: CIPP Context, Input, Process and Produce; CMO context-mechanism-outcome; ToC theory of change

### Contribution analysis

Contribution analysis, one approach within the ‘explanatory generation’ of evaluation (Brousselle & Buregeya, 2018), has been proposed for application in HPE (Moreau & Eady, 2015; Van Melle, Gruppen, Holmboe, Flynn, Oandasan, Frank, et al., 2017). Contribution analysis recognises that outcomes, or lack thereof, can be experimentally observed, yet definitive attribution to a complex program, where a multitude of internal and external factors influence outcomes, is fraught with uncertainty (Mayne, 2001). Instead, CA poses the question of “how and why has the intervention (or component) made a difference, or not, and for whom?” (Mayne, 2019, p.175) to reduce, rather than eliminate, uncertainty. Causality is inferred by generating credible and plausible claims about how a program contributed to observed outcomes through collection and scrutiny of evidence against a theory of change (ToC), thus increasing understanding as to why outcomes were observed (Mayne, 2012, 2019). In contribution analysis, validity is not framed in terms of statistical inference or traditional approaches (Paz-Ybarnegaray & Douthwaite, 2017). Instead, contribution analysis takes a generative approach to causality, supporting internal validity by considering the plausibility of how and why the evaluand, such as programmatic assessment, contributes to intended outcomes.

Contribution analysis posits that program contribution to outcomes is inferred when four key conditions are achieved: (i) development of a reasonable and plausible ToC which articulates how and why a program works to achieve outcomes, (ii) program activities were implemented as set forth in the ToC, (iii) the ToC is confirmed by evidence and is not disproven, and (iv) alternative influential factors are considered and shown not to significantly contribute or the contribution is recognised (Mayne, 2011). First introduced by John Mayne in 1999 to address limitations of experimental evaluation methods in monitoring government programs (Mayne, 2001), contribution analysis has since evolved into a framework for evaluating complex programs in real-world settings (Leeuw, 2023; Mayne, 2019). The evolution of contribution analysis has been categorised into four generations (Budhwani & McDavid, 2017; Dybdal et al., 2010). The first generation focussed on utilising existing data (quantitative and qualitative) for monitoring program performance with nine steps outlined, representing the foundational elements. In the second generation, contribution analysis remained focused on government program monitoring with greater emphasis on operational design, including the integration of a ToC and the consolidation of nine steps into six (Leeuw, 2023; Mayne, 2001). The third generation applied contribution analysis to complex programs, reinforcing the foundational arguments, namely that experimental designs have limited utility in complex situations (Mayne, 2011). The six steps remained largely unchanged, with expanded operational guidelines. The fourth generation, shaped by Mayne (2015) and others (Budhwani & McDavid, 2017), saw growing adoption and highlighted the use of ToC to explore program complexity through activities, mechanisms, outcomes, and context, enhancing the framework’s suitability for evaluating complex real-world programs (Leeuw, 2023).

Contribution analysis evaluates a program using a six-step process, starting with the development of a ToC and followed by aligning this with evidence (Mayne, 2019). The ToC is central to contribution analysis, providing a comprehensive understanding of how the evaluand contributes to change (Brousselle & Buregeya, 2018). While sometimes used interchangeably, ToC differs from program theory. Program theory outlines the logic between evaluand activities and expected outcomes, focusing on underlying assumptions. In contrast, ToC offers a detailed, sequential explanation of how and, importantly, why change occurs within a specific context. Data-informed impact pathways within the ToC represent the change process and the conditions necessary to achieve intended outcomes (Brousselle & Buregeya, 2018; Chen, 2004; Mayne, 2017). The ToC serves as a framework for assessing collected evidence and making contribution claims about evaluand impact. Contribution analysis posits that if the steps and assumptions of a program, as set forth in the ToC, are verified by evidence, it is reasonable to conclude that the intervention contributed to the observed outcomes (Mayne, 2019). Despite the potential in HPE, the application of contribution analysis remains limited, with only one recent study using it to explore the contribution of curricular to health graduate capabilities (Choi et al., 2023).

Contribution analysis is well-suited to evaluate programs situated within and across settings that are characterised by uncertainty and dynamic mechanisms, where outcomes may be attributed to multiple factors some of which may be external to the program (Brousselle & Buregeya, 2018), as applies to programmatic assessment (Govaerts et al., 2022). While programmatic assessment has been shown to achieve certain learner outcomes (Schut et al., 2021), implementation continues to faces challenges (Ryan et al., 2023; Schut et al., 2021) and evidence for health care recipient outcomes beyond the learner remain limited. This knowledge gap becomes increasingly significant as the adoption of programmatic assessment expands beyond health professions education to become the modern paradigm for higher education (Caretta-Weyer et al., 2024; Lodge et al., 2023). Given the need to adopt theory-informed evaluation methods in CBE and the application of contribution analysis to complex programs in other settings (Biggs et al., 2014; Buregeya et al., 2020; Koleros & Mayne, 2019), we aimed to evaluate programmatic assessment using contribution analysis. Specifically, we sought to identify the mechanisms that contributed to the outcomes of programmatic assessment and facilitated sustained implementation. This evaluation also served as a demonstration of how contribution analysis can be applied to HPE.

## Method

### Study context

This study was situated within an interpretivist paradigm and sought to understand how, when, and why programmatic assessment worked by developing a program evaluation framework (a ToC) using data from mixed sources (Rees et al., 2023). All team members held academic roles and brought experience in HPE, with four of us (JJ, CP, RB, SG) having led the design and implementation of programmatic assessment in our respective programs. These experiences shaped our interest in the research question and our choice of a theory-informed approach to explore the mechanisms and contextual factors influencing assessment outcomes. Our positionality informed the development of cause-effect questions and enabled deeper insights during focus groups, as our tacit knowledge of programmatic assessment supported meaningful engagement with participants. We remained reflexive throughout, using team discussions to challenge interpretations, incorporating broader perspectives through the literature review and confirming findings with participants. The research was conducted across four settings, with approval obtained from all institutions (Monash University Research Ethics Committee approval no. 28847, Edith Cowan University Research Ethics Committee approval no. 02561, University of Canberra Research Ethics Committee approval no. 9369, and University of Wollongong Human Research Ethics Committee approval no. 2021/333).

### Terminology for contribution analysis

Terminology is used interchangeably within contribution analysis literature and so we first present terms and definitions (Table 2) (Mayne, 2019). Notably, outputs and outcomes are distinct concepts. Outputs refer to the tangible goods and services derived from program activities, while outcomes denote changes in behaviours and actions (Hall et al., 2021). Within the ToC, outcomes are stratified into capacity change, behavioural change, direct benefit, and well-being change (Mayne, 2017).

**Table 2:** Terms and definitions used in contribution analysis, including alternative terms evident in published literature (Lemire et al., 2012; Mayne, 2015, 2017, 2019)

Term (alternative term(s))	Explanation
Impact pathway (results chain, casual chain, logic model)	A pathway depicting the sequences of steps or events from activities to outcomes.
Assumption	Salient connections, events and or conditions necessary for a link within an impact pathway to function as expected and fulfil program outcomes.
Theory of change	Structured assembly of impact pathways and assumptions presenting how program activities lead to outcomes. Components are activities, outputs, reach and reaction, capacity change, behavioural change, direct benefit, and well-being change.
Activities	Observable actions undertaken as part of the program.
Outputs	Tangible goods and services that directly result from the program activities being undertaken.
Reach and reaction	Identification of the target group (reach) who are intended to receive program outputs and their reaction to the program (reaction).
Capacity change	Changes in knowledge, attitudes (beliefs, opinions, feelings, perspectives), skills (mental and physical ability to use new or alternative practices), aspirations (ambitions, hope, objectives, or desires) and opportunities of the target group who receive or use the program outputs; established using the COM-B model which is the influence of capabilities (C) and opportunities (O) on motivation (M) which are necessary for behaviour (B) change.
Behavioural change	Changes in practice that occur in the target group due to capacity change.
Direct benefit	Improvements in the target group derived from behaviour change.
Well-being change	Long-term accrued improvement in the well-being of beneficiaries who may or may not be the program target group.
External influences	Events and conditions unrelated to the program but that contribute to the realisation of intended outcomes.
Nested theory of change	Additional theories of change which capture a particular component of the complex program.
Contribution claim	Statement(s) presenting evidence and describing the mechanism, or lack thereof, for the contribution the program (or component) makes to observed outcomes.
Contribution story	Central narrative that explains how a program, and components, contribute to the observed outcomes.
Relevant Explanation Finder	Structured framework facilitating critical review of collected data (in step 3 of contribution analysis) against the theory of change.

The following sections describe the six steps of contribution analysis and application in the present study to evaluate programmatic assessment, including the qualitative multi-centre study undertaken in step 3.

### Step 1: set out the cause-effect issue to be addressed

The first step, which describes the problem the evaluand seeks to address and develops cause-effect questions, serves to focus the evaluation (Mayne, 2012) and is usually carried out by a team with program experience (Biggs et al., 2014; Buregeya et al., 2020; Choi et al., 2023; Riley et al., 2018). Following this approach, we (JJ, CP, MH, SG) used our programmatic assessment experiences to develop cause-effect questions. Three of us had led the design and implementation of programmatic assessment at two dietetic programs (JJ at Edith Cowan University (Jamieson et al., 2017), and CP and SG at Monash University (Palermo et al., 2017)) with the fourth researcher having extensive experience in teaching programmatic assessment (MH). We had each evaluated programmatic assessment across the two programs, providing a comprehensive understanding (Dart et al., 2021; Jamieson et al., 2022, 2021). One researcher (JJ) developed cause-effect questions, which were reviewed and agreed upon by the team (CP, MH, SG). These cause-effect questions were: (i) what factors influenced the implementation of programmatic assessment? (ii) what role did programmatic assessment contribute, or not, to intended outcomes? (iii) what conditions were necessary to achieve this contribution? These questions guided the ToC development (step 2) and data collection (step 3) by framing focus group questions.

### Step 2: develop the postulated theory of change and risks to it, including rival explanations

An initial ToC is then iteratively developed often using existing evaluative data (Biggs et al., 2014), stakeholder consultation (Biggs et al., 2014; Delahais & Toulemonde, 2017; Downes et al., 2019; Koleros & Mayne, 2019), and expert discussion (Delahais & Toulemonde, 2012, 2017; Koleros & Mayne, 2019), providing a sound comprehension of program activities, outputs, and intended outcomes (Mayne, 2012). Top-level outcomes (well-being change) are identified first, with impact pathways then constructed in reverse (Riley et al., 2018). A robust and credible ToC is essential, as it provides the framework against which collected evidence is later evaluated (Mayne, 2012, 2019).

We followed several steps to develop the initial ToC for programmatic assessment. The Edith Cowan University dietetic course, which features a programmatic assessment approach, was selected as a case study due to our extensive experience with its design, implementation, and evaluation. The lead researcher (JJ) completed training in program theory and reviewed the contribution analysis literature. The same researcher then conducted a focus group with two faculty staff who had co-designed and implemented the programmatic assessment, provided valuable insight when developing the ToC. Participants were given questions in advance (Online Resource 1), including ToC explanation and key terms, to optimise discussion. The questions, derived from the cause-effect questions in step 1, guided the focus group. During the focus group, the researcher recorded the identified goals, outcomes, activities, assumptions and influencing factors on post-it notes, which were then iteratively ordered, beginning with outcomes and working backgrounds to map causal links (initial mapping is provided in Online Resources 2 item (a)).

After the focus group, one researcher (JJ) used problem analysis to identify and unpack the root causes and consequences of the issue that programmatic assessment sought to address, consulting published research on competency-based assessment. This process articulated the core problem and untangled contributing factors and consequences, producing a Problem Tree verified by co-researchers (CP, MH, SG). Along with the focus group mapping, this was the ToC starting point. Next, the team (JJ, CP, MH, SG) iteratively developed impact pathways by repeated reviewing of the Problem Tree, the focus group mapping, relevant literature, and personal experience. The initial ToC was then refined using the COM-B model proposed and detailed by Mayne (2019) (Online Resource 2 item (b) and (c)). COM-B is a social science model positing the influence of capabilities (C) and opportunities (O) on motivation (M) with all three being critical inter-related conditions needed for behaviour (B) change (Mayne, 2017). COM-B was introduced to contribution analysis as a structured model to explore and explain drivers of behaviour change, enabling robust ToC on which to base inferences (Mayne, 2018, 2019). In the final stage, ToC robustness was evaluated by one researcher (JJ) using Mayne (2017) ToC Analysis Criteria, leading to minor revisions that were reviewed and agreed upon by all other researchers (CP, MH, SG).

### Step 3: gather existing evidence on the theory of change

Step 3 involves gathering evidence to determine the plausibility of the ToC (Riley et al., 2018). Sufficient and rigorous evidence is required to determine if the postulated ToC impact pathways and outcomes occurred as posited (if at all), confirm or challenge assumptions, and identify factors and insufficiencies, all which underpin contribution claims (in step 4) and the contribution story (in step 6) (Mayne, 2012). Evidence can come from existing published and unpublished evaluations (Biggs et al., 2014; Choi et al., 2023), mixed methods research (Buregeya et al., 2020; Hersey & Adams, 2017; Junge et al., 2020), or a combination of methods (Delahais & Toulemonde, 2012, 2017; Downes et al., 2019). Given the limited evaluative data on programmatic assessment at the time of this research, we opted to conduct a multi-centre qualitative study in this step. The following sections detail the methods used in this study during step 3 of contribution analysis.

#### Participant recruitment

In June 2021, two researchers (JJ and CP) delivered a videoconference workshop (Zoom™ Video Communications Inc) on programmatic assessment to 12 of the 16 accredited Australian dietetic programs. The workshop introduced programmatic assessment using Bok et al. (2021) clustering of the 12 principles into three themes. Attendees completed an activity to determine the extent, if any, to which programmatic assessment had been implemented in their programs, enabling the identification of institutions that had adopted the approach. At the workshop conclusion, attendees were informed about the research project and invited to email their completed activity responses to the researchers (JJ and CP) if they were interested in joining as co-researchers. Representatives from the four non-attending universities were contacted by email and given the opportunity to participate, with one reminder sent. Of the 16 programs, eight returned the form, and five were found to have implemented programmatic assessment according to the published principles (Heeneman et al., 2021). One researcher (JJ) then conducted a videoconference with the program representative to verify adherence to the published principles of programmatic assessment through discussion. One university declined to participate due to capacity constraints, while Edith Cowan University, Monash University, University of Canberra, and University of Wollongong joined the study (RB at University of Canberra; JL at University of Wollongong; JJ at Edith Cowan University; and CP, MH, and SG at Monash University). Collaboration with these co-researchers was critical to connect with stakeholders to explore the research question.

From these four universities, three stakeholder groups were recruited for the qualitative study: faculty-employed academics responsible for teaching and assessment, graduates who had completed the program, and workplace supervisors overseeing learner tasks during placement. Inclusion criteria required affiliation with one of the four programs in the prior 12 months. A total of 21 focus groups were conducted with faculty (n = 19), graduates (n = 15), and supervisors (n = 32 participants) across the four universities. Participant characteristics are presented in Table 3. Employed participants (n = 57) worked in community or health promotion (n = 23), hospitals (n = 28), education (n = 22), aged care (n = 5), food service (n = 5), research and development (n = 5), private practice (n = 4), and or disability (n = 1), with employment status including full-time (n = 32), part-time (n = 21), casual (n = 2), and other (n = 2, parental leave, contract).

**Table 3:** Focus group participant demographics by stakeholder group (step 3 of contribution analysis)

	Faculty	Graduates	Supervisors
	(n = 19)	(n = 15)	(n = 32)
**University**			
Edith Cowan University	4	6	1
Monash	5	7	11
University of Canberra	5	1	9
University of Wollongong	5	1	11
Age (years)	47 ± 7 (35–58)	30 ± 10 (22–50)	38 ± 8 (28–57)
**Gender**			
Female	19	12	32
Male	-	2	-
Non-binary	-	1	-
**Employment location**			
Metropolitan	18	5	25
Regional	1	1	4
Rural or remote	-	-	3
Not employed	-	9	-

#### Research setting

All four universities offered a postgraduate dietetic program that mandated 100 days of workplace-based placement where learners undertook authentic activities under the supervision of workplace supervisors. Programmatic assessment was introduced to the dietetic program at Edith Cowan University in 2016, 2018 at Monash University, 2016 at University of Canberra, and 2018 at University of Wollongong. Three of the dietetic programs were post-graduate with variable sized cohorts (20 students at Edith Cowan University, 25 to 40 at University of Canberra, and up to 61 at Monash University). The University of Wollongong had both an undergraduate (30 students) and postgraduate (20 students). Each program adhered to the twelve principles of programmatic assessment (Online Resource 3) as each uniquely utilised an andragogical justified (principle 5) mix of feedback-rich assessment moments (principle 2 and 4), conceptualised as low-stakes data (principle 1 and 6). Learners participated in learning meetings (principle 11) where performance was reviewed and discussed, based on low-stakes data, functioning as intermediate check-in moments and providing an opportunity for individualised remedial action (principle 10). Low-stakes data were collated and reviewed by at least two independent assessors who used consensus building to make a high-stakes decisions which determined program progression and graduation (principle 3, 7 and 9). All four programs applied the Dietitians Australia National Competency Standards (Palermo et al., 2016) as the framework for designing the programmatic assessment and making high-stakes progression decisions (principle 8) and adopted a learner-centred education paradigm where learner agency was promoted (principle 12).

#### Data collection

Co-researchers (JJ, SG, RB, JL) sent the expression of interest email to stakeholders affiliated with their respective programs. The email provided study information and a Qualtrics^TM^ (Provo, UT) survey link for interested individuals to indicate availability for a focus group and provide demographic data (age, gender, geographical location, area(s) of work practice, current workload). Graduates were also contacted using personal messages in LinkedIn to maximise participation. One researcher (JJ) reviewed all Qualtrics survey responses and organised focus groups.

Separate semi-structured videoconference focus groups were held for each program and stakeholder type between October 2021 and February 2023, with interruptions due to the COVID-19 pandemic. Focus groups were conducted separately for graduates, faculty, and supervisors at each university to foster homogenous discussion about programmatic assessment. Each focus group was facilitated by a researcher (JJ or SG) who was not affiliated with the program, with co-researchers (JJ, SG, JL, or RB) familiar to the participants also in attendance. While we recognise that pre-existing relationships could have influenced participants’ sharing (Berger, 2015), we determined that insider knowledge was necessary for contextualising discussions. In mitigation, the facilitator was external to the program under study, providing an outsider perspective.

Nine focus group questions were developed based on the cause-effect questions (developed in step 1) and the postulated ToC (developed in step 2). After the first seven focus groups (two with faculty, two with graduates, three with supervisors), five additional questions were added to capture topics not explicitly covered but relevant to the contribution analysis. These additional questions explored views on high-stakes progression decisions, relationships, managing learner underperformance, and employability (Online Resource 4). Focus groups were between 24 and 81 minutes, were audio recorded, and transcribed using Otter.ai (AISense). One researcher (JJ) reviewed and edited all transcriptions for accuracy.

#### Data analysis

Using the Framework Analysis Method (Gale et al., 2013), two researchers (JJ and SG) abductively coded three focus groups (one from each stakeholder group) in an iterative approach referring to the ToC and research questions. The researchers then discussed and agreed on an analytical framework, grouping codes into categories reflecting the ToC components. The initial framework included eight codes and 46 sub-codes, each with a definition and illustrative quotation. The framework and all transcribed focus groups were then entered into NVivo™ Version 12 (QSR International) for subsequent analysis by one researcher (JJ). During analysis, four new sub-codes were added relating to professional competency standards, client outcomes, preparing learners for practice, and improved competency-based assessment practices. These changes were reviewed and agreed to by a second researcher (SG). The final framework is given in Online Resource 5. Themes were iteratively mapped to the ToC, leading to revision. Data mapping involved documenting the source by stakeholder group (graduates, supervisors, faculty) and the frequency of the data, as required for step 4 of contribution analysis to verify the ToC and develop the contribution claims. Researchers (JJ, SG, RB, JL) reviewed the results, and discussions in two meetings confirmed agreement on the analysis and consistency across stakeholders and universities. Key findings were then carried forward into step 4.

### Step 4: assemble and assess the contribution claim, and challenges to it

In contribution analysis, evidence from step 3 is analysed to identify and scrutinise impact pathways and influencing factors. Influencing factors are contextual conditions that enable or hinder outcomes (Lemire et al., 2012). The Relevant Explanation Finder, introduced by Lemire et al. (2012) and adapted by others (Biggs et al., 2014; Buregeya et al., 2020), structures data analysis and support the construction of contribution claims (Delahais & Toulemonde, 2012). A contribution claim asserts the presence (or absence) of change, the contributing links(s), and influencing factor(s) (Delahais & Toulemonde, 2012). Contribution claims are iteratively mapped to links in the ToC, which is then revised (Biggs et al., 2014; Mayne, 2012). A preliminary contribution story, including a revised ToC and supporting narrative, is then developed and may be reviewed by stakeholders to identify additional evidence to strengthen the evaluation (Delahais & Toulemonde, 2012; Mayne, 2012).

One researcher (JJ) used Microsoft Excel to create the Relevant Explanation Finder, incorporating an Evidence Analysis Database (Delahais & Toulemonde, 2012). The structure and application of the Relevant Explanation Finder are well-described elsewhere (Biggs et al., 2014; Delahais & Toulemonde, 2012; Lemire et al., 2012). The researcher systematically assembled and critically assessed the synthesised data according to each column heading, with a second researcher reviewing (SG). Iteratively, the researcher (JJ) used the data compiled in the Relevant Explanation Finder, frequently referencing step 3 data synthesis, to develop contribution claims and revise the ToC. Following Delahais and Toulemonde (2012) approach, each contribution claim included a mechanism label, a description, and any further actions required such as ToC revision or further evidence collection. All researchers then met and discussed the contribution claims and revised ToC, reaching consensus through discussion. The final result was nine contribution claims with nine assumptions, two external influences, and three threats to programmatic assessment (given in Online Resource 6).

### Step 5: seek out additional evidence

In step 5, additional data is gathered to strengthen the links within the ToC and enhance the credibility of the contribution story (Mayne, 2012). Various approaches to this step are reported, including merging it with earlier steps (Downes et al., 2019; Hersey & Adams, 2017), expert review (Choi et al., 2023; Delahais & Toulemonde, 2012, 2017), further targeted data collection (Koleros & Mayne, 2019), and accessing secondary data sources (Koleros & Mayne, 2019; Riley et al., 2018).

As all components of the ToC and contribution claims were substantiated by evidence collected in step 3 and further data collection was constrained by the study timeline, we opted to conduct a stakeholder (from step 3) review and gather secondary data from published evaluations of programmatic assessment, which had increased since the research began. For the stakeholder review, one researcher (JJ) created a 17-minute video presenting the ToC and contribution story. This video was reviewed, and minor edits made by two other researchers (MH and SG) before recording. The final video was emailed to all participants from step 3, inviting them to view it and provide feedback via a Qualtrics^TM^ survey. The survey asked respondents to confirm they had watched the video, identify their stakeholder group (graduate, supervisor, faculty), identify three key learnings, comment on whether the findings reflected their experience with programmatic assessment, specify any areas needing clarification, and suggest topics they would like to explore further. Participants had two weeks to respond. After which, one researcher (JJ) compiled and reviewed the survey data. Feedback indicated that the ToC and contribution story accurately represented responding participants’ experiences with programmatic assessment and was clear. As such, no further modifications were made to the ToC based on participant feedback.

A literature review was then conducted to strengthen the ToC and enhance the robustness and transferability of the findings. One researcher (JJ) searched electronic databases (MEDLINE (Ovid), Embase (Ovid), Web of Science, Scopus, and Cumulative Index to Nursing and Allied Health Literature Plus (EBSCO)) on 23 February 2023 using the term “programmatic assessment” in the title. Inclusion criteria were empirical evaluations of programmatic assessment across any discipline, written in English, and published after 8 December 2019. This date was chosen as Schut et al. (2021) had published a literature review on programmatic assessment, synthesising research up to that point. The search yielded 407 publications which were imported into Covidence (Veritas Health Innovation). Covidence was used to identify and remove 279 duplicates, leaving 128 publications for title and abstract screening. At title and abstract screening, 41 publications were excluded as did not address programmatic assessment or did not provide empirical evidence, leaving 87 publications for full text review. Eleven articles met the inclusion criteria with one (Jamieson et al., 2021) excluded as we (JJ, CP, MH, SG) authored the paper and findings had been incorporated into the ToC in step 2. The remaining ten articles (Baartman et al., 2023, 2022; Dart et al., 2021; Jamieson et al., 2022; de Jong et al., 2022; Roberts et al., 2022; Ross et al., 2023; Schut et al., 2020, 2021; Torre et al., 2022) were included. The same researcher (JJ) extracted data (publication year, title, aim, setting, methods, participants, results) into an Excel worksheet (summarised in Online Resource 7). This data was then mapped to the contribution claims, using colour coding to indicate the source. In an iterative process, the extracted data and ToC were reviewed, and contribution claims were revised. These revisions were reviewed by another researcher (CP), and the final updates to the ToC and contribution claim were finalised. After revisions, there were seven contribution claims and eleven assumptions, with no change to the three threats and two external influences. One proposed mechanism was discarded due to insufficient evidence (Online Resource 6, contribution claim 9).

### Step 6: revise and strengthen the contribution story

Revising, strengthening and presentation the contribution story is the final step (Mayne, 2012), often involving critical review by a steering group or expert panel (Choi et al., 2023; Delahais & Toulemonde, 2012, 2017; Downes et al., 2019). Based on the collection, analysis, and integration of additional data in step 5, one researcher (JJ) revised and finalised the contribution story, which was reviewed by all researchers with no further amendments indicated.

## Results

The ToC and contribution story representing the impact pathways necessary for effective and sustained programmatic assessment were developed.

### Theory of change for programmatic assessment

**Fig. 1:**
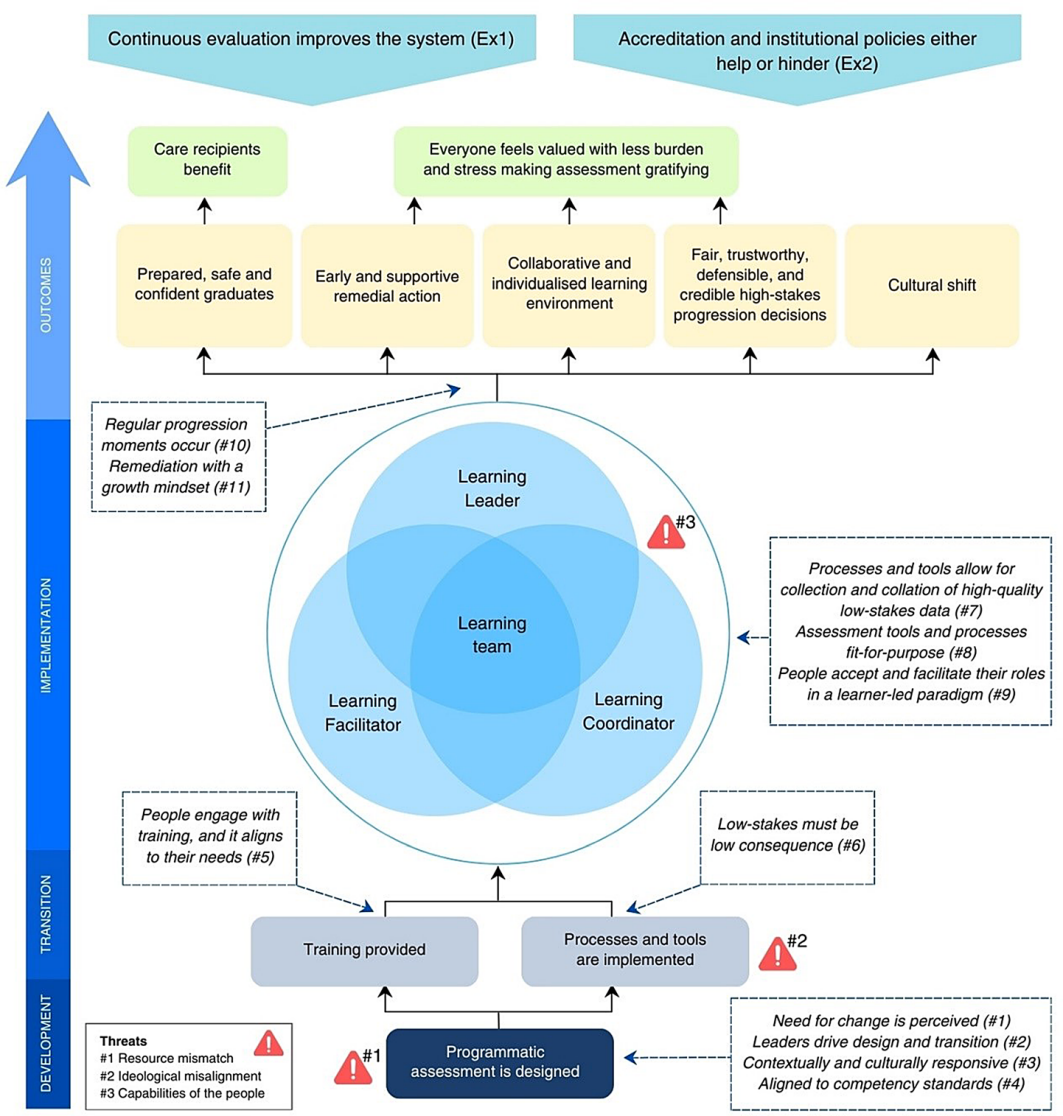
Theory of change for programmatic assessment developed using contribution analysis, presenting the impact pathway from development to outcomes, and includes threats (red triangles), assumptions (in dashed textboxes), and external influences (Ex1 and Ex2). ^a^ Abbreviations: Ex External influences

**Fig. 2:**
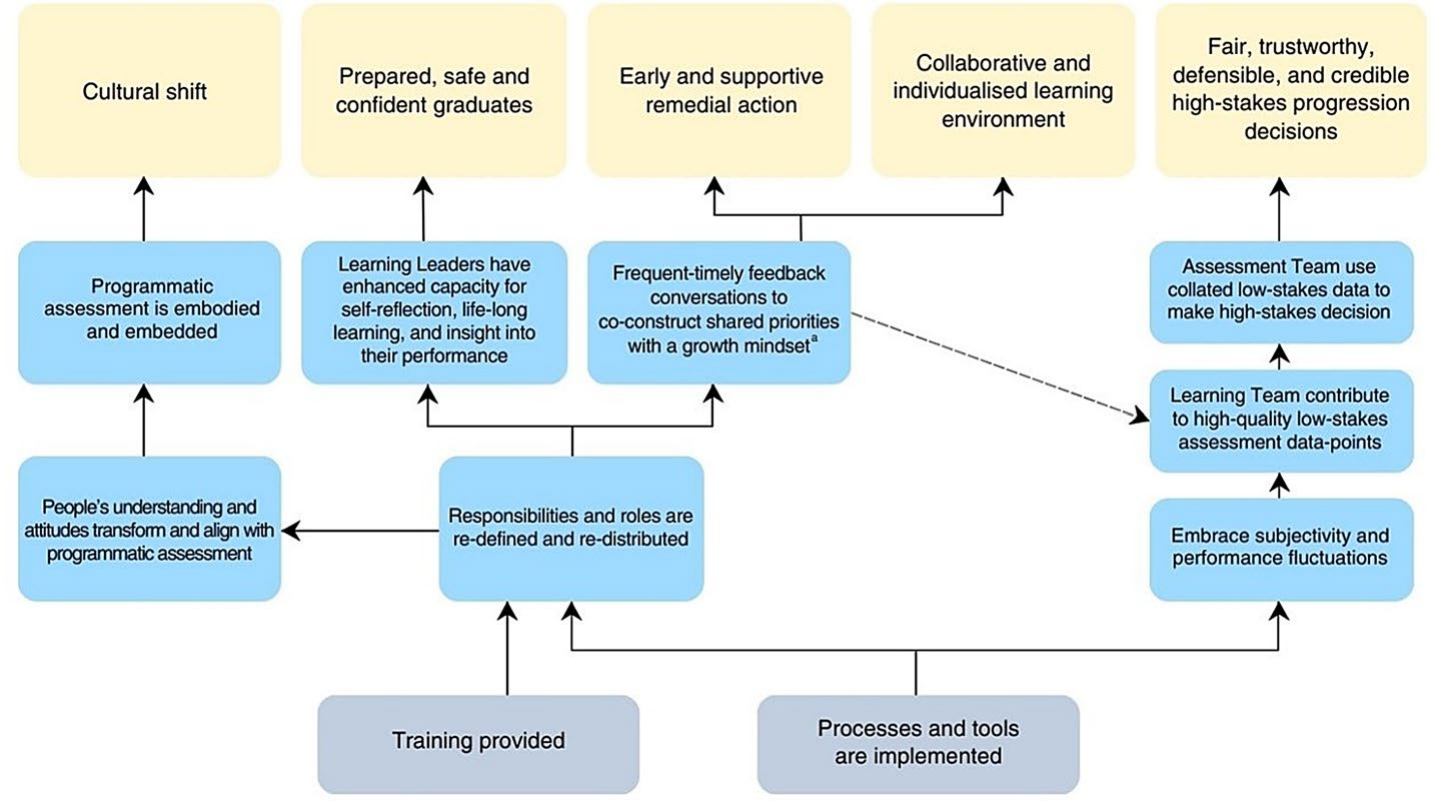
First nested impact pathways within the theory of change for programmatic assessment that outlines how outputs lead to outcomes. ^a^ Indicates location of the second nested impact pathway (detailed in Fig. 3)

**Fig. 3:**
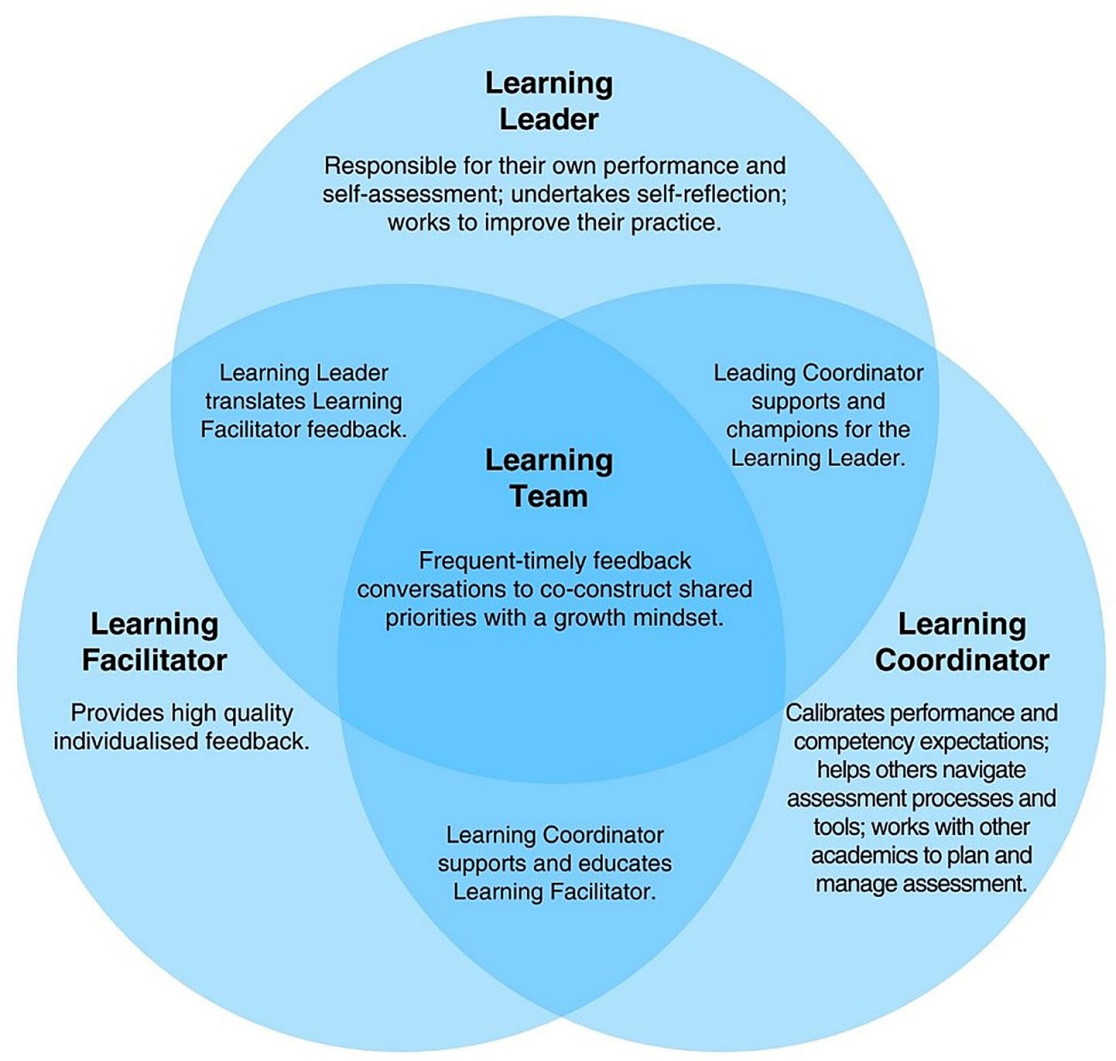
Second nested impact pathways within the theory of change for programmatic assessment that illustrates relationships between stakeholders

The ToC articulates the impact pathways, assumptions, threats, and external influences enabling successful and sustained programmatic assessment (Fig. 1). Impact pathways are the sequential steps leading from activities to outcomes (Mayne, 2019). Following the contribution analysis process (Mayne, 2015), the ToC presents the initial *activity* of designing a programmatic assessment, leading to the *outputs* of training and implementation. This is followed by the *reach and reaction,* with *capacity* and *behaviour change* illustrated in a Venn diagram to capture their complexity. These are further unpacked in the nested impact pathways shown in Figure 2 and 3. The *direct benefit* to the target groups (prepared, safe and confident graduates; early and supportive remedial action; collaborative and individualised learning environment; fair, trustworthy, and credible high-stakes progression decisions; and cultural shift) culminate in *well-being* changes to the people involved in programmatic assessment and health care recipients. These represent the outcomes for programmatic assessment observed across the multi-centre qualitative study (step 3) and literature review (step 5).

The data revealed three key teams essential to programmatic assessment, the (i) design team, (ii) learning team, and (iii) assessment team. The design team was comprised of knowledgeable and capable leader(s) who guided the design and implementation of programmatic assessment. The learning team was comprised of three stakeholders: the learning leader, learning facilitator, and learning coordinator who worked cohesively to create a psychologically safe individualised learning environment. The assessment team held responsibility for high-stakes progression decisions and sat outside the other teams, although individuals could belong to multiple teams and have discrete responsibilities.

### Contribution story for programmatic assessment

The following section gives the contribution story for programmatic assessment which details the impact pathways presented visually in the ToC (Fig. 1). The contribution story was developed by following the six steps of contribution analysis which built upon the case study (step 2) with qualitative research (step 3) and a literature review (step 5). The contribution story integrates contribution claims, assumptions, threats, and external influences into a narrative. Contribution claims provide the explanation and evidence for, or lack, impact pathways within the ToC. Assumptions are salient conditions necessary for the link within the ToC to function as expected, and for the intervention to achieve outcomes. We identified eleven assumptions for programmatic assessment that are highlighted in the following section and presented in Fig. 1. Threats, indicated in Fig. 1, were identified from the data as notable constraints to programmatic assessment. External influences are events and conditions unrelated to programmatic assessment but that impact the realisation of the intended outcomes.

#### Leaders drove transition

The perceived need for change, arising from internal needs or external developments, was the precursor to designing a programmatic assessment for a particular context (assumption #1). Knowledgeable, strategic, and collaborative leaders were needed to garner support and establish alliances, creating opportunities and momentum. These leaders brought together and oversaw a design team, kept the vision in focus, and built-in sustainability (assumption #2). The design team used the principles of programmatic assessment as over-arching guides to shape a unique programme that was authentic and responsive to the context and culture in which it was implemented (assumption #3). All principles of programmatic assessment were sufficiently flexible and versatile and well suited to this purpose. The design team uses relevant professional standards or learning outcomes as the foundation for developing the programmatic assessment (assumption #4). Challenges and tensions were encountered during the design process, and leaders (and the design team) skilfully used compromise to overcome these whilst adhering to the over-arching principles of programmatic assessment. This starting design point for programmatic assessment could be undermined by resource mismatch (threat #1). Programmatic assessment requires substantial input from stakeholders during development and initial implementation and there can be a tendency towards over-assessment. This may exceed resource capacity, namely stakeholders and their time, which can overwhelm people during implementation and compromise success. In turn, this negated the desired behaviours such as feedback giving and seeking, degraded the feedback quality, and undermined trust in programmatic assessment. A well-considered balance was needed to optimise the often-insufficient resources, enhanced by continuous evaluation which could refine programmatic assessment over time (external influence #1).

#### Training developed capabilities of the people

As part of the programmatic assessment, formal (i.e., workshops) and informal training (i.e., educative conversations) on assessment literacy (focussed on programmatic assessment rationale and concepts) and application of processes and tools was provided to everyone. Training occurred initially, during design, and ongoing. This created a common understanding and language amongst people and equipped them with knowledge and capabilities to enact roles and responsibilities, facilitating implementation as intended by the design team. This built capacity in the people and contributed to the sustainability of the programmatic assessment. It was vital that people had access to and engaged with training that was aligned to their needs (assumption #5).

#### High-quality low-stakes data-points underpinned high-stakes progression decisions

The purposefully designed programmatic assessment included fit-for-purpose tools used as intended (assumption #8). The people engaging with the programmatic assessment recognised that learner performance fluctuated and that interpretations of standards varied, allowing subjectivity to be embraced. This approach highlighted the value of their contribution to generating low-stakes assessment data. For low-stakes assessments to function as assessments for learning, they must genuinely be perceived as low consequence (assumption #6). A multitude of high-quality, feedback-rich, low-stakes data about a learner were obtained longitudinally from different perspectives. Processes and tools were in place to collect and collate this low-stakes data for interpretation by the assessment team (assumption #7). The collated evidence gave a holistic and balanced picture of a learner’s pattern of performance.

The assessor was situated outside the learning team and had access to the low-stakes data for each learner, making them best positioned to undertake high-stakes progression decisions as part of an assessment team. Through a consensus building process, this separate assessment team used the consistency and congruency of collated low-stakes data to make a high-stakes progression decision which was viewed as being fair, trustworthy, defensible, and credible. This process overcame challenges in managing differing opinions and disagreement. Critical to this process, was all people feeling that their contribution was valued. As assessment data was co-created and responsibility shared, the burden on each person was minimised which alleviated stress and made assessment gratifying for learners, faculty, and supervisors.

#### A learning team was fostered

Programmatic assessment re-defined and re-distributed responsibilities and tasks with three stakeholders revealed as being critical: the (i) learning leader (ii) learning facilitator, and (iii) learning coordinator (presented in Fig. 3). Learners were the leaders who gained responsibility by being given, and accepting, responsibility for assessment by translating feedback, undertaking self-reflection, and engaging in feedback discussions. Within this learner-led paradigm, the learner was given ownership of and empowerment over their assessment. Workplace-based supervisors were learning facilitators, focused on observation, teaching, and providing quality, individualised feedback. By supporting learners in their self-reflection and assessment, supervisors gained insight into the learner’s perspective such as their feelings, circumstances, thinking which contributed to positive shifts in roles and strengthened relationships. Faculty staff (or faculty-employed coaches) were learning coordinators who had a supportive and educative role which enabled others to enact and feel comfortable in their responsibilities. Learning coordinators also managed learner-led remedial actions, calibrated performance expectations, advocated for learners, and collaborated with other faculty to oversee assessment. These three roles (learning leader, learning coordinator, and learning facilitator) coalesce into a collaborative learning team that adopted a growth mindset and engaged in frequent, timely feedback conversations to co-construct shared priorities and expectations, all centred on supporting the learner. The result was a collaborative, productive, psychologically safe, and individualised learning space where constructive performance conversations were transparent and easier for all. For learners, this helped overcome self-doubt and self-criticism which reduced stress, while faculty and supervisors felt more involved and invested, leading to greater satisfaction. Yet this division of roles and responsibilities could be compromised by ideological and philosophical misalignment, which often arose when introducing programmatic assessment and challenging the status quo. Resistance to programmatic assessment stemmed from (i) disruption to existing authority and power dynamics, (ii) unfamiliarity and uncertain with the approach, and (iii) misalignment between personal or organisational beliefs, values, and practices and those of programmatic assessment. For most, training and exposure facilitated understanding and acceptance, leading to a reduction in resistance. Although for some, ideological dissonance and resistance remained (threat #2).

Key stakeholders within programmatic assessment needed to understand and accept their roles within the learner-led paradigm with success contingent on their buy-in and capabilities (assumption #9). Learning leaders who struggled to engage due to factors such as lack of motivation, insight, self-reflection skills, language barriers (particularly relevant for narrative-based assessments), or underperformance, could hinder the effectiveness and increase the workload for others. Learning facilitators who provided insufficient or low-quality feedback or felt excluded from high-stakes progression decisions could also compromise effectiveness. Additionally, extraneous demands (work or personal) on any stakeholders could hinder the success of programmatic assessment (threat #3).

#### Progression moments enabled early detection of issues and psychologically safe, learner-led remedial action

Members of the learning team participated in regular progression reviews where learner performance and interpretations were discussed collaboratively, fostering a shared mental model (assumption #10). This enabled early identification of underperformance, allowing for timely, individualised, and supportive remedial action focussed on growth (assumption #11). Learners had agency in their remedial action, which contributed to their sense of psychologically safety.

#### Learners became prepared, safe, and confident graduates

Learners used programmatic assessment processes and tools to gain insight and provide evidence for progression. The learner-led approach equipped learners with the skills, knowledge, and attributes necessary for practice, fostering self-reflective lifelong learners with the ability to identify and work within the bounds of their abilities while seeking support when needed. Graduates who had been assessed within programmatic assessment described themselves, and were perceived by faculty and supervisors, as confident and well-prepared to enter the workforce as safe and effective practitioners. This was seen by learners, faculty and supervisors as benefiting the recipients of their care.

#### Programmatic assessment became the status quo

Over time, attitudes for most stakeholders evolved to align with programmatic assessment, with the principles and ideologies becoming embodied by people and embedded within the infrastructure. This transformation instigated a culture shift that contributed to the sustainability and normalisation of the practice, particularly feedback.

#### External influences: evaluation and institutional polices

Evaluation and quality improvement were essential to evolving and enhancing programmatic assessment, ensuring it continued to meet the needs of all stakeholders. Accreditation and institutional policies were powerful influencers in shaping programmatic assessment with support being critical to success. Yet there could be a lag between early adopters and accreditation and institutional policies which was a hinderance. The principles of programmatic assessment were sufficiently flexible to accommodate discipline and context specific nuances, to an extent. Ultimately, change was enabled when accreditation and institutional policies aligned with the principles and ideology of programmatic assessment.

## Discussion

Applying the six steps of contribution analysis, we evaluated programmatic assessment to identify the impact pathways from inception to implementation, and described the contribution to outcomes, considered from the perspectives of stakeholders and published literature. We identified the mechanisms that contributed to the outcomes of programmatic assessment, demonstrating how contribution analysis can be applied in health professions education. We found that programmatic assessment, when enacted in accordance with the principles, made a noticeable contribution to an improved learning environment leading to psychologically safe learning spaces and relationships, as well as enabling credible high-stakes progression decisions which prepared graduates for practice. Ultimately, people experienced less stress and derived gratification from assessment and there was the perception that clients benefited from better prepared learners. The impact pathways for these contributions were clear and plausible, informed by data which underwent scrutiny and demonstrated congruency, enabling the verification of the ToC. External factors, namely institutional and accreditation requirements, were shown to influence programmatic assessment and their contribution was recognised within the ToC. In accordance with contribution analysis, the findings of this research are considered probabilistic rather than definitive proof (Mayne, 2012).

### Implications of findings

Application of theory-informed evaluations in HPE is nascent. Van Melle et al. (2021) put forward a proposed logic model for competency-based medical education which resonated with our own findings. Agreement was observed in processes and outputs (leadership, tool development, user preparation, educational alignment) which contributed to learner-related outcomes (feedback, agency, remedial action, multiplicity of data) and ultimately to practice and client outcomes (readiness and transition to practice, enhanced client outcomes). External factors were similar (institutional structures and role of continuous evaluation) as was inclusion of growth mindset theory. Alternative approaches to constructing the two models such as purpose, methods, setting, data sources, and scope likely contributed to differences, and are not unexpected given the dynamic and context-sensitive nature of HPE (Van Melle et al., 2021). Congruency between our work and that of Van Melle et al. (2021) provides a deepening understanding of mechanisms enabling competency-based assessment and the role played by programmatic assessment.

As with Van Melle et al. (2021), we found that the adoption of a growth mindset by the learning team was critical to programmatic assessment. Mindset theory, the perceived malleability of one’s capabilities, is recognised as a factor in how people engage with learning (Dweck, 2019; Sahagun et al., 2021; Yun-Ruei & Catanya, 2022). Those with a growth mindset view personal attributes as improvable, while those with a fixed mindset see them as inherent and unchangeable (Sahagun et al., 2021). People with a growth mindset perceive challenges as opportunities for improvement, which has been shown to enhance psychological well-being and academic performance (Sahagun et al., 2021; Williams & Lewis, 2021; Wolcott et al., 2021; Yun-Ruei & Catanya, 2022). Mindsets are fluid and shaped by the environment. Education can foster a growth mindset by promoting learner agency (Yun-Ruei & Catanya, 2022), using mastery-focussed goals (Richardson et al., 2021; Williams & Lewis, 2021; Wolcott et al., 2021), offering regular low-consequence feedback (Sahagun et al., 2021), emphasising formative assessment (Williams & Lewis, 2021), reducing reliance on numeric grades (Richardson et al., 2021), and framing mistakes as a part of learning (Sahagun et al., 2021; Wolcott et al., 2021). Educators, including supervisors, coaches, teachers, are instrumental in modelling this mindset through performance-focussed feedback, encouraging learner reflection, and engaging in open conversations (Williams & Lewis, 2021; Wolcott et al., 2021). These strategies mirror the intent of programmatic assessment and were observed in our findings which, through assessment design, fostered a growth mindset to produce reflective and adaptable learners. Our findings suggest that programmatic assessment may promote learner motivation, a key driver of learning and psychological wellbeing (Kusurkar et al., 2023).

Preparing learners to meet the health needs of the community is the paramount outcome for CBE (Frank et al., 2010). Paradoxically, this poses a challenge to evaluators and evidence for attainment is limited (Van Melle et al., 2021; Wong et al., 2012). While we report some data suggesting improved client outcomes, these were based on perceptions of a learner’s capabilities as seen by others, rather than directly from the client’s experience. Although the ToC recognises client outcomes as the outcome of programmatic assessment, the certainty of contribution remains limited. Determining the impact of HPE on client outcomes is a critical step and further research, using theory-informed frameworks is recommended to deepen understanding. Given the challenges of linking HPE to measurable client outcomes, longitudinal evaluation methods and approaches that engage clients in the design are needed. These methods, underpinned by program theory, are likely to offer more meaningful insights into how HPE contributes to improved health outcomes (Haji et al., 2013; Oandasan et al., 2020). Learner data derived from programmatic assessment may need to shift towards a greater reliance on collective competency assessment with evidence of health impact.

Despite growing enthusiasm for programmatic assessment, its viability within a resource constrained higher education sector remains a concern. Scalability within large cohorts and adoption in established programs are challenges (Ryan & Judd, 2022; Torre et al., 2021), and there are barriers to sufficiently resourcing an individualised learning approach (Torre et al., 2021). Assessment resources are finite and each decision requires balancing benefits and costs, considered as financial, educational, or otherwise (Cleland et al., 2020). Emerging evidence suggests that authentic, peer-based assessment tasks may yield improved outcomes (Kusurkar et al., 2023), which begs the question as to what role, if any, knowledge-based summative assessment has in HPE and if cost savings can be made. Implementers of programmatic assessment must make pragmatic decisions informed by ‘useful knowledge’ which tells them how and why (Baartman et al., 2022; Brown et al., 2015; Sandars, 2018). Our findings build on previous work (Baartman et al., 2022; Torre et al., 2021) by sequencing the critical steps and revealing the influential factors and threats. This knowledge can practically be used to prioritise resources for maximal outcomes and support creative solutions to design dilemmas, guided by educator priorities (Cleland et al., 2020). Although a component of the assessment utility index (van der Vleuten, 1996), little is known about the cost-effectiveness of programmatic assessment or HPE broadly (Cleland et al., 2020). We demonstrated that programmatic assessment enabled early learner-led remedial action and minimised assessment burden, hypothesised to produce future savings due to the hidden associated costs (Ellaway et al., 2018; Torre et al., 2021). Further research using economic concepts is needed to explore how educators evaluate the costs and consequences of programmatic assessment. For example, does investment in learner-led processes, low-stakes assessment, and training lead to later gains in learning management and reduced attrition, as proposed by others (Ellaway et al., 2018; Steinert, 2013)? Ultimately, an approach combining satisfaction and sufficiency, what Cleland et al. (2020) refers to as ‘satisficing’, may be required to implement programmatic assessment with finite resources. Understanding the true cost of programmatic assessment, a balance between design choices, resource utilisation, and long-term benefits, has the potential to enhance feasibility and support widespread adoption.

### Application of contribution analysis in health professions education

We found that the structured contribution analysis framework facilitated rigorous interrogation of probable causality between programmatic assessment activities and outcomes, enabled by data collection and synthesis, and stakeholder discourse. The cornerstone was the ToC, with nested theories key to unpacking and presenting the complexities in a way that avoided oversimplification (Frye & Hemmer, 2012). While others have proposed and used logic models (Choi et al., 2023; Van Melle et al., 2021), we found the ToC to be comprehensive which facilitated deeper exploration. This was enhanced by thorough application of the six steps of contribution analysis.

While contribution analysis can be time and labour intensive, making it unsuitable for all situations (Mayne, 2019; Van Melle, Gruppen, Holmboe, Flynn, Oandasan, Frank, et al., 2017), we found the investment justified given the paucity of evaluative evidence on programmatic assessment and its increasing adoption. Although expertise in contribution analysis is essential, the process can be broken into manageable steps that can be incrementally applied by teams with varying levels of expertise. Reflecting on our experience, there is value in considering efficiency, especially given the calls for greater application of theory-informed evaluation within HPE (Moreau & Eady, 2015). Although we retrospectively constructed the ToC, we advocate for an ex-ante approach in step 1 and 2 that engages stakeholders as a steering group during program development to develop a postulated ToC. This upfront engagement clarifies how the program is intended to work, fostering a unified understanding for enhanced delivery and providing a blueprint for subsequent evaluation (Frye & Hemmer, 2012). Investing in this front-end work may mitigate workload burdens and embed continuous evaluation to improve practices (Mayne, 2019; Oandasan et al., 2020). This approach may also offer the opportunity to engage stakeholders more purposefully over time, allowing meaningful involvement in the co-construction of the ToC which may further enhance the plausibility of the findings. We also see potential to build on our efforts and those of others by publishing program theories within HPE which could contribute to targeted data gathering in step 3. Sharing these published program theories and guides would be valuable for those with limited experience in theory-informed evaluation, offering examples and insights. This could assist the collective construction of knowledge regarding the impact of HPE on client outcomes which, although vital, is challenging to determine due to the multitude of influencing factors unfolding over long timeframes (Hall et al., 2021; Van Melle, Gruppen, Holmboe, Flynn, Oandasan, Frank, et al., 2017). Such actions would streamline contribution analysis processes for widespread adoption and beget HPE outcome evidence.

Experimental evaluation, emblematic of a scientific paradigm, is not only impractical in HPE from a design and cost perspective, but the pursuit of an objective truth is misaligned with inherent multifaceted complexity of educational practice (Frye & Hemmer, 2012; Grant & Grant, 2023; Palermo et al., 2021). Increasingly in HPE, we recognise truth as multiplicitous and dynamic, constructed through an individual’s socio-cultural context, reflected in the ontological stance of relativism (Rees et al., 2020, 2023; Van Melle, Gruppen, Holmboe, Flynn, Oandasan, Frank, et al., 2017). Although the philosophical underpinnings of contribution analysis have limited exploration, there is alignment with critical realism through the acknowledgement and integration of multiple perspectives within the ToC, achieved through iterative stakeholder engagement and by promoting research methods beyond quantitative (Brousselle & Buregeya, 2018; Leeuw, 2023). Contribution analysis enables multiple impact pathways to be identified, providing evidence often required for HPE practice decisions, for example, what program leaders need to implement to achieve programmatic assessment success. Importantly, we are recognising the need to construct HPE knowledge using different, theory-informed evaluation approaches, across different settings (Allen et al., 2022; Van Melle, Gruppen, Holmboe, Flynn, Oandasan, Frank, et al., 2017).

### Limitations

Our contribution analysis involved robust data analysis, adherence to steps, research team reflective discussions and recurrent engagement with stakeholders strengthened the methods and findings (Oandasan et al., 2020). We recognise that the inclusion of dietetic programmes in step 3 limits transferability. To address this, we vetted the dietetic programmes against the principles of programmatic assessment, provided a rich context description, and intentionally integrated evidence derived from alternative disciplines (in step 5) to enhance transferability. Given the significant influence of contextual variables such as culture, resources, and governance structures, these factors would further mediate the transferability of these findings to other settings. The four dietetic programmes included in step 3 had small to medium learner cohorts, and as suggested (Ryan et al., 2023), there are likely to be distinct challenges associated with larger cohorts and other disciplines. We recognise the limitation of narrowing the scope to programmatic assessment implemented in workplace-based learning. Increasing adoption of programmatic assessment across the higher education sector will underscore the need for research to explore implementation in undergraduate and university-based settings. There is also much to be learnt from exploring situations where programmatic assessment implementation was attempted and achieved either in part or not at all. Importantly, ToC should not be considered static, it is a modifiable theory responsive to emerging and alternative data (Oandasan et al., 2020). We anticipate, and look forward to, future research which applies theory-informed evaluation methods to inform our collective understanding of programmatic assessment.

## Conclusion

Using contribution analysis, we have evaluated programmatic assessment to construct a ToC which articulates the mechanisms from activities to outcomes. Leverage and risk points have been illuminated which educators can apply to navigate contextually responsive and successful manifestations of programmatic assessment across diverse settings. This research contributes to the health professional education community understanding of CBE outcomes and drives improvements in practice.

## Electronic supplementary material

Below is the link to the electronic supplementary material.

Supplementary Material 1

Supplementary Material 2

Supplementary Material 3

Supplementary Material 4

Supplementary Material 5

Supplementary Material 6

Supplementary Material 7

## Data Availability

No datasets were generated or analysed during the current study.
