# Supplementary material for: An evaluation of programmatic assessment across health professions education using contribution analysis
Source: Adv Health Sci Educ Theory Pract. 2025 Jun 4;31(1):211–38. doi: 10.1007/s10459-025-10444-5 (PMC12929344; doi:10.1007/s10459-025-10444-5)
Supplement: Supplementary file 1 — Supplementary Material 1 [file 10459_2025_10444_MOESM1_ESM.docx]

**Online Resource 1.** Focus group questions used with faculty staff (n = 2) during step 2 of contribution analysis to evaluate programmatic assessment.

| 1. In your opinion, what is the ultimate goal of the Edith Cowan University Master of Nutrition and Dietetics assessment programme? 2. How does the Edith Cowan University Master of Nutrition and Dietetics assessment programme achieve this goal? 3. We now need to consider the ordering of the outcomes on a time continuum from short to medium term (or proximal to distal) relative to the long-term goal(s).    1. How would you arrange the identified outcomes?    2. Why have you ordered the outcomes in this way? 4. What are the program activities, actions or initiatives that led to the short term outcomes? 5. Assumptions are external conditions that must exist, or that are taken to exist, for program outcomes to be achieved. Assumptions often remain hidden to the users of a program.    1. What assumptions exist for this programmatic assessment to achieve its goal(s)?    2. At what points in the theory of change does the assumption exert its influence?    3. Why have you identified the assumption? What is your rationale?    4. What risks are associated with the assumption? 6. What are the external influences and contextual factors that influence the programmatic assessment? 7. Are there any changes you would like to make to the theory of change? 8. What evidence can be collected to evaluate the theory of change? |
| --- |
