# Supplementary material for: An evaluation of programmatic assessment across health professions education using contribution analysis
Source: Adv Health Sci Educ Theory Pract. 2025 Jun 4;31(1):211–38. doi: 10.1007/s10459-025-10444-5 (PMC12929344; doi:10.1007/s10459-025-10444-5)
Supplement: Supplementary file 2 — Supplementary Material 2 [file 10459_2025_10444_MOESM2_ESM.docx]

**Online Resource 2.** Theory of change versions (a, b, and c) for programmatic assessment created throughout the contribution analysis process.

**External and contextual factors**

- University policies (e.g., grading schemas)
- Working Group approach to developing the programmatic assessment in the Master of Nutrition and Dietetics at Edith Cowan University
- Facilitator or Educator supports student during their placement and competency development
- Dietitians Australia National Competency Standards
- Internal funding to support the design the programmatic assessment in the Master of Nutrition and Dietetics at Edith Cowan University

Graduate capable practitioners who can meet the needs of the community now and into the future.

**Assumption**

- National Competency Standards meet current and future community and workforce needs.

Identification of competence and not competent learners.

**Assumption**

- Competence differentiable possible with evidence.

**Assumptions**

- Assessors have expertise to assess competencies.
- Sufficient evidence to determine student competence.

Learners who demonstrate the Dietitians Australian National Competency Standards.

Demonstrated self-reflection and life-long learning.

Multiple university-based assessors contributing to high-stakes decisions.

High-quality feedback provided to the learner.

**Assumptions**

- Learners engage with assessment (their ability and motivation).
- Stakeholders engage with roles and transition.
- Essential moments are captured in feedback.
- Stakeholders have skills to provide feedback.
- Programmatic assessment is best practice.

Delineation of roles within assessment.

Multiple opportunities for formative assessment and feedback.

Learner-centred.

Fit-for-purpose assessment with timely high-quality frequent feedback and reduced repetition.

**Assumptions**

- Assessment is learner centred.
- Training is adequate and targeted to the needs of stakeholders.

**Activities**

- Assessment instruments that are learner-led
- Training of stakeholders (practitioners, learners, university staff)
- Multiple assessment instruments
- Same assessment instruments across placement settings
- One portfolio for assessment of competence

(a) First version of the theory of change for programmatic assessment based on the stakeholder focus group undertaken in step 2 of contribution analysis.

**ACTIVITIES** (actions)

ECU MND WIL assessment redesigned according to the programmatic principles of best practice CBA and implemented.

**OUTPUTS** (direct goods and services)

1. Multiple new (or revised) assessment instruments (“fit for purpose”)
2. Delineation of user [who are the users? Is the term assessor?] roles within the assessment programme
3. User [who are the users? Is the term assessor?] training

**REACH AND REACTION** (the target group and their response)

- Learners engage with the assessment programme
- WBAs engage with the assessment programme

**Well-being change** (long-term cumulative improvement)

Needs of the community now and into the future are met by graduates.

**Direct benefit** (improvements in target group or beneficiaries)

- Learners are capable of life-long learning.
- Credible and defensible assessment decisions made about learner performance.
- Identify competent and not competent learners.

**Behavioural change** (changes to actual practice)

A. Learners receive high quality feedback

A. Learners receive feedback from multiple sources

B. Workplace-based assessors adopt teacher role

B. Learners adopt self-assessment role

- Learners progress in their competence development
- Learners transition their learning across the placement settings

**Capacity building** (changes in knowledge, skills, attitudes, aspirations, and opportunities in the target group)

Learners:

- Knowledge: identification of learner strengths and weaknesses; understand the programmatic assessment.
- Attitude: assessment supports learner competence development; positive about the programmatic assessment.
- Skills: ability to self-reflect and self-learn.
- Aspirations: successful (pass) placement; enter the workforce.
- Opportunities: advance skills; employment.

Workplace-based assessors engage with the programmatic assessment:

- Knowledge: best practice assessment; effective feedback; understand the programmatic assessment.
- Attitude: positive about the programmatic assessment; stress related to placement is reduced.
- Skills: effective teachers.
- Aspirations: effective placement work-based assessor; see learners progress and graduate.
- Opportunities: reduce assessment burden.

**Reach and reaction** (target group and their response)

- Learners engage with assessment.
- Workplace-based assessors engage with assessment.

**Outputs** (direct goods and services)

A Multiple new (or revised) assessment instruments that are “fit for purpose”.

B Delineation of stakeholder roles with programmatic assessment.

C Stakeholder training.

**Activities** (actions)

The Edith Cowan University Master of Nutrition and Dietetic assessment for placement is redesigned according to the principles of programmatic assessment.

(b) Second version of the theory of change for programmatic assessment with the integration of the problem tree analysis in step 2 of contribution analysis.


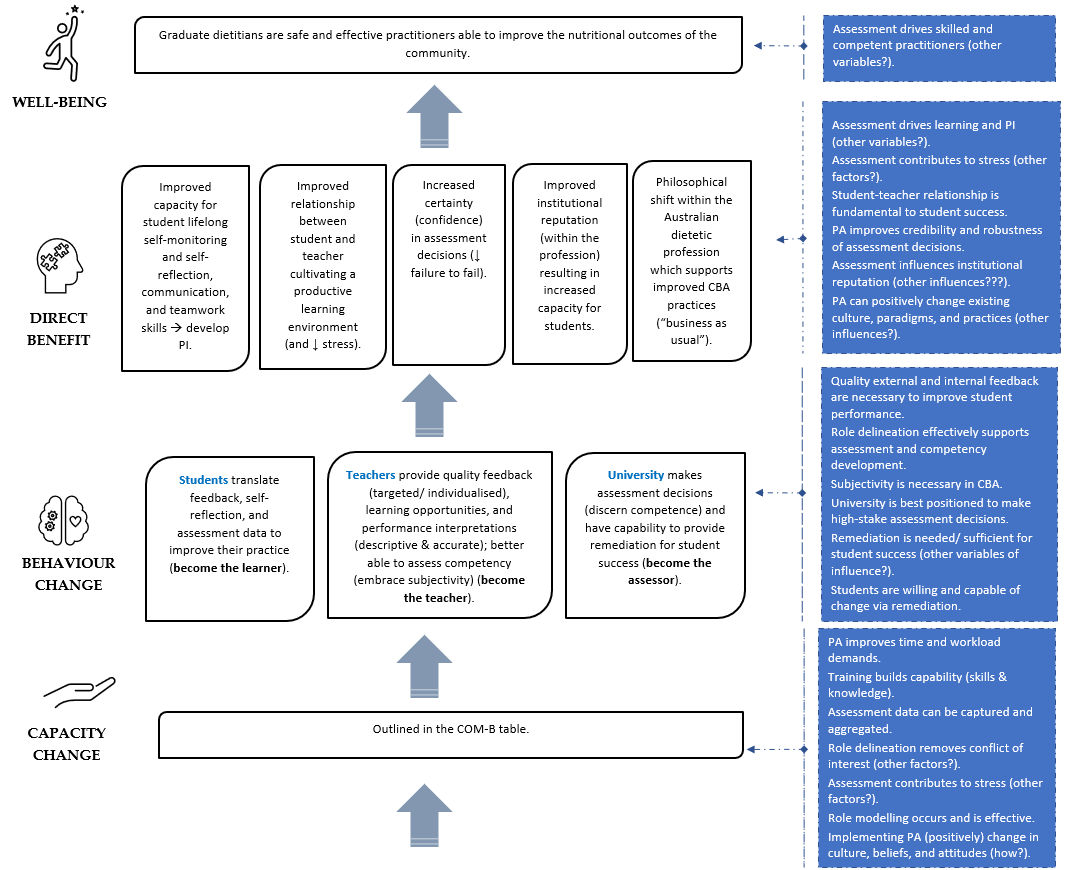

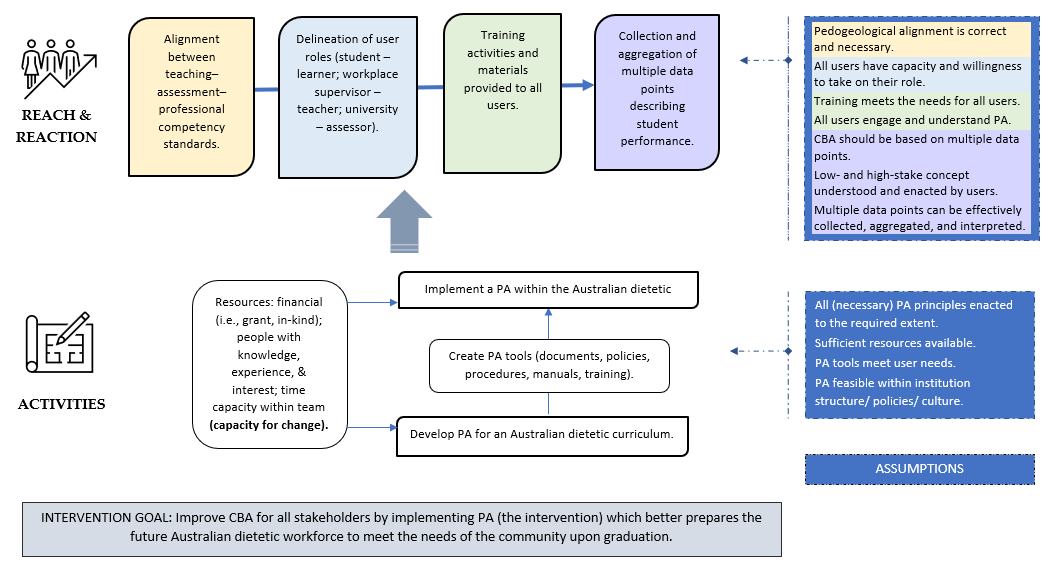


(c) Third version of the theory of change for programmatic assessment with the inclusion of COM-B model (capability – opportunity – motivation for behaviour change) developed in step 2 of contribution analysis.
