# Supplementary material for: An evaluation of programmatic assessment across health professions education using contribution analysis
Source: Adv Health Sci Educ Theory Pract. 2025 Jun 4;31(1):211–38. doi: 10.1007/s10459-025-10444-5 (PMC12929344; doi:10.1007/s10459-025-10444-5)
Supplement: Supplementary file 3 — Supplementary Material 3 [file 10459_2025_10444_MOESM3_ESM.docx]

**Online Resource 3.** Adherence to the principles of programmatic assessment for the four Australian dietetic programs included in the qualitative study in step 3 of contribution analysis (adapted from Heeneman et al., 2021).

| **Programmatic assessment principles** | **Edith Cowan University** | **Monash University** | **University of Canberra** | **University of Wollongong** |
| --- | --- | --- | --- | --- |
| 1. Every (part of an) assessment is but a data-point | **Yes**  Multiple assessments during placements (e.g., self and supervisor performance appraisals; work outputs including reports, medical documentation, and case studies; self-reflection; learning goals; client and stakeholder experiences) capture learner development and capability; no single assessment determines progression decision. | **Yes**  Multiple low-stakes assessment moments (e.g., self, peer and supervisor appraisals and feedback; work outputs including authentic reports and medical documentation; learning development plans; critical reflections) which capture learner development. | **Yes**  Multiple low-stakes assessments (e.g., work samples: research manuscript/s, reports, simulations, case-studies, presentations; multisource feedback: student performance appraisal moderated by supervisor’s performance appraisal, clients and stakeholders feedback; critical reflections; learning development plan) which capture learner development. | **Yes**  Multiple low-stakes assessments during placements (e.g., self and supervisor performance appraisals; work outputs including reports, client resources, medical documentation, relevant stakeholder presentations; self-reflection; learning goals; client and stakeholder experiences) capture learner development and capability; no single assessment determines progression decision. |
| 2. Every data-point is optimised for learning by giving meaningful feedback to the learner | **Yes**  Performance assessments are narrative-based, undertaken first by the learner and then others, and discussed together at Reflective Progress Meetings. All assessments designed to elevate feedback. Learners reflect across all learning, then develop individualised learning goals. | **Yes**  Formative and summative assessments contain opportunities for reflection and feedback | **Yes**  Students self-assess their own practice against criteria / entrustable professional activities /learning goals using a narrative to justify their judgement that includes demonstrated outcomes/capabilities and identified learning needs. This is moderated by their supervisor and discussed at a student-led Reflective Progress Meeting that is used to develop an individualised learning plan. | **Yes**  Low-stakes assessments are learner-led and contain opportunities first for self-reflection and appraisal, then incorporation of collaborative feedback, and culminating in guided progress discussions at relevant time points. Individualised goals are developed by learners. |
| 3. Pass/ fail decisions are not given on a single data-point | **Yes**  No progression decisions made on a single assessment, rather low-stakes assessments (as listed above) collated into portfolio by learner and entirety of data is reviewed for high-stakes progression decision. | **Yes**  Overall assessment decisions based on several data-points and discussed with stakeholders across settings. Decisions are made in consideration of all data points at regular student progress meetings. | **Yes**  No progression decisions made on a single assessment, rather low-stakes assessments (as listed above) collated into portfolio by learner and entirety of data is reviewed for high-stakes progression decision. | **Yes**  Low-stakes assessments collated into portfolio. No progression decisions made on a single observation or single assessment evidence. Holistic portfolio review for high-stakes progress decision. |
| 4. There is a mix of methods of assessment | **Yes**  Variety of assessments including narrative, checkboxes, self-reflection, work artefacts; all mapped by faculty to professional competency standards to ensure alignment with learning outcomes. | **Yes**  Variety of assessment tools applied including supervisor direct observations using narrative and checkboxes, learner reflections, work artefacts and portfolios. | **Yes**  Variety of assessments including narratives, checkboxes, self-reflection, work artefacts; all mapped by faculty to professional competency standards to ensure alignment with learning outcomes. | **Yes**  Variety of tools applied for assessment. Most are qualitative in nature to elicit valuable self-reflection and meaningful feedback to strengthen connection to professional competency standards and learning outcomes in a range of practice settings. |
| 5. The method chosen should depend on the education justification for using that method | **Yes**  Faculty and industry supervisors selected and designed assessments aligned with authentic practice, which undergo ongoing review by experienced health professions education faculty. | **Yes**  Assessment methods developed with consideration of assessment settings, assessors, and stakes. Assessment methods consider utility for all stakeholders including supervisors, patients, learners and faculty. | **Yes**  Faculty with experience in health professions education determine tools with consideration of assessment settings, assessors, and stakes. | **Yes**  University coordinators with experience in health professions education determined tools. Industry supervisors invited to share authentic practice work activities for integration in assessments that are regularly reviewed by experienced faculty. |
| 6. The distinction between summative and formative is replaced by a continuum of stakes | **Yes**  Low-stakes assessments do not result in progression decisions but rather provide feedback to the learner on their performance and support remedial action. Continuum of stakes language and ideology adopted in training of learners and supervisors. | **Yes**  Continuum of stakes with formative and summative assessment being distinct in some settings. Low stakes assessments provide feedback to learners to support ongoing development. Continuum of stakes language and ideology adopted in training of learners and supervisors. | **Yes**  Low-stakes assessments do not result in progression decisions but rather provide feedback to the learner on their performance and support remedial action. Continuum of stakes language and ideology adopted in training of learners and supervisors. | **Yes**  Continuum of stakes language and ideology adopted in training of learning and supervisors. Focus on supporting remedial action is highlighted across settings. |
| 7. Decision-making on learner progress is proportionally related to the stake | **Yes**  Individual low-stakes assessments direct learning and remedial action. Compilation of these assessments, along with learner reflections, in a portfolio informs progression decisions for course completion and graduation. | **Yes**  High-stakes decisions (progression and completion) based on collated low-stakes data-points. Compilation of these assessments, along with learner reflections, informs progression decisions for course completion and graduation. | **Yes**  Individual low-stakes assessments direct learning and remedial action. Compilation of these assessments, along with learner reflections, in a portfolio informs progression decisions for course completion and graduation. | **Yes**  High-stakes decisions (progression and completion) based on collated low-stakes data-points (the portfolio). See previous comments on contents of portfolio. |
| 8. Assessment information is triangulated across data-points towards an appropriate framework | **Yes**  Individual low-stakes assessments designed for, and mapped to, Dietitians Australia National Competency Standards. Faculty mapping ensures each competency is captured in multiple assessments for triangulation during high-stakes progression decisions; learners are not required to map to individual competencies.  Assessors iteratively read learner’s portfolio to triangulate data and confirm competence. | **Yes**  Individual low-stakes assessments designed for, and mapped to, Dietitians Australia National Competency Standards. Faculty mapping ensures each competency is captured in multiple assessments for triangulation during high-stakes progression decisions; learners also take responsibility for their learning by identifying the competencies that are brought into play. | **Yes**  Individual low-stakes assessments designed for, and mapped to, Dietitians Australia National Competency Standards. Faculty mapping ensures each competency is captured in multiple assessments for triangulation during high-stakes progression decisions; learners also take responsibility for their learning by identifying the competencies that are brought into play to complete the whole tasks / entrustable professional activities in their e-portfolio. | **Yes**  Multiple assessment data-points; applied the Dietitians Australian National Competency Standards.  Assessors iteratively read learner’s portfolio to triangulate data and confirm competence. |
| 9. High-stakes decisions are made in a credible and transparent manner, using a holistic approach | **Yes**  Two assessors independently review learner’s portfolio. All assessors then convene to discuss each learner. If both assessors agree, the outcome stands. In cases of disagreement or uncertainty, a third assessor, not involved in earlier discussions and decisions, reviews the portfolio. Assessors then come together to make a final decision using consensus building. | **Yes**  High stakes assessment tasks are performed by experienced assessors with input sought from other stakeholders where required. Single assessment tasks for borderline and underperforming learners are performed by two independent assessors. Meetings held with faculty to view and discuss assessments with input from learners and placement educators. | **Yes**  High stakes decisions made independently by panel of experienced assessors (two faculty, clinical educator, and Indigenous expert). Ten percent of cohort also moderated by external academic. Judgements shared, areas of disagreement /uncertainty identified for clarification and discussion with the learner in order to reach a consensus decision. | **Yes**  Meetings held with university placement coordinator to view and discuss assessments and portfolio with input from learners and placement educators |
| 10. Intermediate review is made to discuss and decide with the learner on their progression | **Yes**  Reflective Progress Meetings held with learner, supervisor, faculty to reflect on progression at predetermined intervals. No high-stakes progression outcomes made at these meetings, rather they serve to facilitate shared understanding and support remedial action where required. | **Yes**  Reflective Progress Meetings with learner, supervisor, faculty to reflect on progression are held at regular intervals. No high stakes progression outcomes are made at these meetings, rather used to develop learning plan. Remediation action and support provide by faculty as required. | **Yes**  Reflective Progress Meetings held with the learner and supervisor to reflect on and support progression at pre-determined intervals. No high stakes progression outcomes are made at these meetings, rather used to develop learning plan. Remediation action and support provide by faculty as required. | **Yes**  Reflective Progress Meetings with learner, supervisor, university placement coordinator to reflect on and support progression.  No high-stakes progression outcomes made at these meetings, rather they serve to facilitate shared understanding and support remedial action where required. |
| 11. Learners have recurrent learning meetings with (faculty) mentors/ coaches using a self-analysis of all assessment data | **Yes**  Reflective Progress Meetings occur at regular pre-determined intervals and attended by learner, supervisor, and faculty. Learners first discuss performance holistically, informed by self-analysis of assessment information, then as a team, all parties discuss progress. | **Partial**  Learners use self-analysis of assessment data and have regular meetings with supervisors. Only those highlighted as needing further support engage in recurrent learning meetings with faculty. | **Yes**  Moderated self-assessment completed by learner in consultation with supervisors. This is used to develop learning plan. Those highlighted as needing further support engage in recurrent learning meetings with faculty. | **Yes**  Learner driven self-analysis of assessment data during portfolio progress meetings in consultation with university placement coordinator. |
| 12. Programmatic assessment seeks to gradually increase the learner’s agency and accountability for their own learning through the learning being tailored to support individual learning priorities | **Yes**  Learner-led paradigm fostered through formal training, conversations, and role-modelling by faculty. Separate workshops equip learners and supervisors with assessment knowledge, skills, and ideology. Learners supported to led discussions about own learning with agency increased, in response to learner confidence. Learner-led Reflective Progress Meetings support individualised learning plans. | Learner-led learning paradigm with learner undertaking self-assessment and leading supervisor review meetings, supported by individualised learning plans. Separate workshops equip learners and supervisors with assessment knowledge, skills, and ideology. Learners supported to led discussions about own learning with agency increased, in response to learner confidence. | **Yes**  Learner-led paradigm, knowledge and skills scaffolded through formal training (learner and supervisor), education resources (exemplars/videos of assessment tools and processes), conversations and role modelling (including Reflective Progress Meeting [students] and Portfolio Panel [supervisors]). | **Yes**  Learner-led learning paradigm with learner undertaking self-assessment and driving their learning including devising individualised strategies for progress, supported by weekly action plans. |
