# Supplementary material for: An evaluation of programmatic assessment across health professions education using contribution analysis
Source: Adv Health Sci Educ Theory Pract. 2025 Jun 4;31(1):211–38. doi: 10.1007/s10459-025-10444-5 (PMC12929344; doi:10.1007/s10459-025-10444-5)
Supplement: Supplementary file 4 — Supplementary Material 4 [file 10459_2025_10444_MOESM4_ESM.docx]

**Online Resource 4.** Focus group questions, inquiry logic, and use with each type of stakeholder for the multi-centre qualitative study in step 3 of contribution analysis.

| **Focus group questions** | **Focus group question inquiry logic and, where relevant, application to the theory of change** | **Stakeholder type** | | |  |
| --- | --- | --- | --- | --- | --- |
|  |  | **Faculty** | **Graduates** | **Supervisors** |  |
| **Initial focus group questions** | | | | | |
| What does the term ‘programmatic assessment’ mean to you? | Exploring comprehension of programmatic assessment and creating shared understanding between participants and researchers for the focus group discussion. | ⦁ | ⦁ | ⦁ |  |
| Thinking back to your dietetic studies, can you recall the types of assessments you had for your placements? | Prompting recollection of programmatic assessment experiences to enhance focus group discussion and provide insight into participant interpretations of experiences. |  | ⦁ |  |  |
| What are your thoughts on how the [institution] assessment tasks determined your competence or your ability to practice as a dietitian? | Exploring trustworthiness and credibility of the programmatic assessment; verified, expanded, and/ or refuted theory of change impact pathways. |  | ⦁ |  |  |
| In your opinion, what is the purpose or goal of (programmatic or competency-based) assessment? | Exploring and identifying intended outcomes of assessment; verified, expanded, and/ or refuted theory of change impact pathways. | ⦁ | ⦁ | ⦁ |  |
| Do you think the (programmatic or competency-based) assessment at [institution] has achieved these goals? If yes, which goal/s do you think have been achieved? | Exploring perceived achievement, or not, of intended outcomes of programmatic assessment; verified, expanded, and/ or refuted theory of change impact pathways. | ⦁ | ⦁ | ⦁ |  |
| What do you think has helped, or hindered, achievement of these goals at [institution]? | Ascertain how outcomes are, or are not, achieved for programmatic assessment; verified, expanded, and/ or refuted theory of change impact pathways. | ⦁ | ⦁ | ⦁ |  |
| What do you perceive as the key and necessary components of (programmatic or competency-based) assessment? | Ascertain critical and conditional steps for the achievement of programmatic assessment outcomes; identification of assumptions in the theory of change and verified, expanded, and/ or refuted impact pathways. | ⦁ | ⦁ | ⦁ |  |
| Are there any changes you would make to the (programmatic or competency-based) assessment for [institution]? If yes, what are the changes and why would you make them? | Probing to identify challenges and participant priorities for programmatic assessment from the perspective of graduates and supervisors; verified, expanded, and/ or refuted theory of change impact pathways and assumptions. |  | ⦁ | ⦁ |  |
| The next two questions related to changes to the programmatic assessment at [institution].  (a) What changes have been made to the programmatic assessment and why?  (b) Are there any further changes you want to/ would want to make and why? | Probing to identify challenges and participant priorities for programmatic assessment from faculty perspective, recognising their agency in designing the system; verified, expanded, and/ or refuted theory of change impact pathways and assumptions. | ⦁ |  |  |  |
| How would we know if (programmatic or competency-based) assessment has worked? What would be the evidence? | Exploring tangible outcomes for programmatic assessment; verified, expanded, and/ or refuted theory of change impact pathways. | ⦁ | ⦁ | ⦁ |  |
| Can you comment on the ongoing sustainability (or implementation) of the programmatic assessment at [institution]?  (a) In your opinion, what would or would not contribute to (successful) sustainability?  (b) What could improve the (successful) sustainability? | Exploring the conditional factors that contribute to programmatic assessment; incorporated into the theory of change to support successful and sustainable implementation. | ⦁ |  | ⦁ |  |
| What do you think was the most important thing that was said here today, during the focus group? | Providing participants an opportunity to reflect on, and emphasis aspects of the discussion; considered in refining the theory of change. | ⦁ | ⦁ | ⦁ |  |
| **Additional focus group questions^** | | | | | |
| Have you had difference of opinion with other supervisors or [institution] regarding an assessment outcome? If yes, what was your experience? | Exploring challenges to programmatic assessment and how these are handled; incorporated into the theory of change. | ⦁ |  | ⦁ |  |
| Has programmatic assessment changed your relationship to faculty staff/ supervisors/ students? If yes, can you describe the change?  If not, how could it? What would need to happen? | Exploring the implications to relationships and the role relationships have in programmatic assessment; verified, expanded, and/ or refuted theory of change impact pathways. | ⦁ |  | ⦁ |  |
| Can you think of an example when a student did not pass or “did not go well”? What happened? | Exploring challenges to programmatic assessment and how these are handled; incorporated into the theory of change. | ⦁ |  | ⦁ |  |
| Has the organisation for which you work employed graduates from [institution] recently? If yes, can you describe their transition to work. | Seeking evidence for observed outcomes of programmatic assessment; building certainty of impact pathways in the theory of change. |  |  | ⦁ |  |
| If you are working as a dietitian, what are your thoughts on how the assessment prepared you for the workforce? | Seeking evidence for observed outcomes of programmatic assessment; building certainty of impact pathways in the theory of change. |  | ⦁ |  |  |

^^^ Focus group questions were added after the first seven focus groups (two with faculty, two with graduates, three with supervisors).
