# Supplementary material for: An evaluation of programmatic assessment across health professions education using contribution analysis
Source: Adv Health Sci Educ Theory Pract. 2025 Jun 4;31(1):211–38. doi: 10.1007/s10459-025-10444-5 (PMC12929344; doi:10.1007/s10459-025-10444-5)
Supplement: Supplementary file 5 — Supplementary Material 5 [file 10459_2025_10444_MOESM5_ESM.docx]

**Online Resource 5.** The framework analysis used for coding data in the multi-centre qualitative study in step 3 the contribution analysis (codes and sub-codes have been presented with an illustrative quotation and mapped to the relevant component in the theory of change).

| **Code** | **Sub-code** | **Explanation** | **Illustrative quotation** | **Relation to ToC** |
| --- | --- | --- | --- | --- |
| Defining programmatic assessment | Collated evidence | Compilation of performance evidence that gives a holistic picture of a student’s competence. | *…that combination of materials, the input in the portfolio, so you hear multiple voices along their journey of how they're performing using or multiple skills and tasks along the way.* (Faculty, Edith Cowan University) | Activities & Assumptions |
|  | Comparison to professional standards | Using assessment to measure student performance against relevant professional standards. | *…assessing your ability to fulfil a certain set of standards or practices that aligned with being competent in the field that you’re studying.* (Graduates, Monash University) | Reach & Reaction |
|  | Longitudinal | Multiple assessment moments, points and moments occurring over time; with the purpose to monitor, and reflect on, student progression. Holistic approach. | *… assessment over a period of time, not just one point in time. So we'll look at how say a student is progressing.* (Faculty, Edith Cowan University) | Activities & Assumptions |
|  | Multiple perspectives | Different stakeholders providing their interpretation of student performance (usually over time). | *…not one piece of assessment that is sitting in silo but everyone contributing to piece a picture together to support the student going forward.* (Faculty, Monash University) | Activities & Assumptions |
|  | Unfamiliar | Participant is not familiar with the term programmatic assessment or cannot define. | *This is the first I've heard of programmatic assessment is your explanation today.* (Supervisor, Monash University) | Activities & Assumptions (knowledge) |
| Purpose and outcome of programmatic assessment | Client (community) outcomes | Goal of assessment is to graduate health professionals who can meet the needs of clients (individual, groups, populations, systems). This can be speculation by participants or a belief that the assessment system has enabled graduates to achieve this outcome. | *…they're demonstrating skills that will enable an effective education to a patient. So yeah, I think that's an important component of assessment.* (Supervisor, Monash University) | Well-being |
|  | Confident graduates | Building confidence to enable students to enter the profession feeling well equipped and capable. | *…having like the confidence to go out into the workforce, that would be a big like, that it's works.* (Graduate, Edith Cowan University) | Direct benefit |
|  | Credible & trustworthy | Assessment of competence is credible, defensible, trustworthy, equitable and fair (accepted) and accurate. | *…the way that competency is captured because it's quite robust, well very robust with the paperwork they do, if that gives face to the industry that the dietitians that are coming out as graduates are demonstrating a multitude of skills and competencies.* (Faculty, Edith Cowan University) | Direct benefit |
|  | Drives (individualised) learning | Assessment aligns to both the professional standards and the key individual student needs/ areas to develop (i.e., teaching and assessment are tailored). | *…the conditional passes, where we actually look at the individual, we look at the competencies, we see where the gaps are, and we come up with further assessment that's really tailored…it's very student centred…*(Faculty, Monash University) | Behaviour change |
|  | Employment | Use of graduate employment outcomes to determine success of assessment practices. | *…if they want to continue in a dietetic stream after graduating.* (Graduate, Edith Cowan University) | Direct benefit |
|  | Enjoyment & satisfaction | Stakeholders derive enjoyment/ satisfaction/ purpose from their role and work. Burden and stress are minimised. | *…you don't get that many rewards being a practitioner all the time, too. But that's a nice one to have that sort of that satisfaction or that feeling of contribution and making a difference….that's also very uplifting.* (Supervisors, University of Canberra) | Capacity change |
|  | Feedback provision | Students are provided meaningful feedback. | *…towards the end of placement - probably because I was being sort of…self-critical - and then hearing from my supervisor “no, you're good” was like, oh, okay, I'm good and then having…a….visible way to measure your progress, and then to…look back on it as well and to see those areas is yeah, it was pretty useful.* (Graduate, Monash University) | Behaviour change |
|  | Improving CBA | Transitioning to programmatic assessment is an opportunity to ‘fix’ problems in CBA. | *Maybe we're trying to get rid of some of the things that we…were talking about in terms of those barriers.* (Faculty, University of Wollongong) | Capacity change |
|  | Improved relationships & teams & roles | Developing relationships between stakeholders and enhancing enactment of roles and tasks. | *…you learn more about the student and how they're feeling and you know…their insight… really endears you to the student…I feel like I'm part of that journey towards competency, whereas previously, I think it was just about all they have to fill in. I have to fill in this paperwork. It's a hurdle to them…It's done. Move on.* (Faculty, Edith Cowan University) | Capacity change  Behaviour change |
|  | Lifelong learners | Develops students into graduates who are self-directive, self-reflective, insightful (scope of practice) and lifelong learners. | *…having the reflections in the portfolio…pushed me to become more reflective and that kind of encouraged me to just automatically continue to reflect - because even though my portfolio is finished - I found that such a useful tool to better myself and improve on areas that needed improving…if I didn't have those reflective pieces in the portfolio, I'm not sure if…I would reflect as much as I do now, because that kind of got me into the habit.* (Graduate, Edith Cowan University) | Direct benefit |
|  | Philosophical shift | Philosophical shift in the values, beliefs, and practices in the profession that aligns with best practice. | *…there's been a real change and a real shift - even some in some of our sites that perhaps we had more concerns with - they've even come on board. And they've been much better to deal with, I think, since we've changed the way we're doing things.* (Faculty, Monash University) | Behaviour change |
|  | Prepared for practice & competent | Prepares and determines if students are at the graduate standard and can integrate into the workforce and meet the needs of the community. May also include preparing students for placement or WIL. This includes identify students strengths and passions/ interest areas; apply knowledge to practice; conceptualising safe practice; practices and thinking used by stakeholders to determine competence (i.e., job ready). Facilitates transition to employment. | *the other thing about knowing it's working, is potentially about how prepared the students are when they arrive at placement as well. And I must say that we're getting a lot of feedback to say that the students are very well prepared.* (Faculty, Monash University) | Well-being |
|  | Progression & remediation | Processes and systems in place to determine/ capture/ describe student performance (often relative to professional standards) and/ or detect issues or concerns early before escalation and provide collaborative-timely opportunities to remediate, monitor and review progress and, if needed, provide additional and/ or targeted learning opportunities for development. | *…you see that in the portfolio at the end when you look at the students descriptions of how they've…gone from, say, a beginner through to being competent…when you look at their portfolio as a whole and you see that progression, and you see the…development in their ideas, and…their reflections become more meaningful, I feel like it is that progression demonstrates the value of programmatic assessment.* (Faculty, Edith Cowan University)  *I really was careful with the way I worded it because I wanted her to think the learning plan was not us, like, you know, earmarking here as a potential problem student but more of a, you know, there's one particular area you're having a lot of problems with. I want you to be upfront with your supervisor on day one and say I need more support here… help you to be in a position to get through and she was really positive about it.* (Faculty, Monash University) | Behaviour change  Capacity change  Activities |
|  | Reputation | The university/ profession/ particular stakeholders are held in higher esteem by others. | *…might actually be one of the unis that we have the greatest relationship with them. It's because of the team involved and the support that they can offer… when we do feedback…you see the next year that changes have been made to reflect that back. Yeah, I think that helps maintain a good relationship.* (Supervisor, Monash University) | Direct benefit |
|  | Student reflection & insight | Reflection is a normalised learning tool, and it provides others (supervisors or university) with insight into student decisions and actions. There is transparency in discussions and decisions. | *it provides the opportunity for students to reflect in a really comprehensive way at…various time points in their placement, and I think the way [university] has structured those reflective practice meetings for each different placement…provides students…structure so they have a timeline…I think that's, that's really helpful.* (Faculty, Edith Cowan University) | Behaviour change |
| Helpers | Assessment tools, processes & systems | Adaptable, malleable, flexible assessment systems and principles. Fit-for-purpose instruments that capture meaningful stakeholder interpretations of student performance; purposefully utilised within a system or process. | *the structure needs to make sense as well. And we need to be really clear about why we're actually asking for that information.* (Faculty, Edith Cowan University) | Activities (tools)  Reach & Reaction  Capacity change |
|  | Authentic assessment | Flexible and malleable assessment that reflects and aligns with the real world. | *Even if you do the same placement as someone else with the same projects…you're going to have a different experience…having the flexibility in the assessment gives you a really good understanding of not only what you're competent at and what you think you need to work on, but also what you like and what you want to do as your career path.* (Graduate, University of Wollongong) | Activities  Capacity change |
|  | Consistent standards & expectations | Professional standards, shared mental model, and expectations are created and used for consistent assessment. Provide opportunities to create a shared mental model and consistent standards, often through discussion. | *I do recall a few times having differences of opinions with the students…I felt (a) quite supported by uni and being able to discuss that, but I also felt quite supported by the process….My process was to…go back to…the competencies or the EPAs, and I actually found doing that really helpful for me in having that conversation with the student.* (Faculty, University of Canberra) | Reach & Reaction  Capacity change |
|  | Growth mindset | Assessment is viewed as a learning opportunity with a growth, rather than fixed, mindset. Includes self-criticism. | *how can I support this person with their growth and insight into what they know.* (Supervisor, University of Canberra) | Capacity change  Behaviour change  Direct benefit |
|  | Holistic system | Viewing or talking about assessment as a ‘big picture’ and a system. | *it wasn't till I sort of got to the end of it, that I could see the whole big picture of how all the little bits and pieces have set me up to be able to practice as a dietitian.* (Graduate, Edith Cowan University) | Activities |
|  | Knowledgeable & capable users | Users are involved and equipped, through training or other means, to effectively participate in programmatic assessment. This includes the ability to have conversations to create a shared mental model for performance expectations and practices. | *as time progressed, and we were a bit clearer about why we wanted all that information. And I think became better at conveying that to the student. And I think then the process just became easier for everybody.* (Faculty, Edith Cowan University) | Activities |
|  | Leadership | Direction, support, and advocacy for programmatic assessment. | *There needs to be a vision that it's better say, than traditional assessment. And obviously, that takes leadership and commitment.* (Faculty, Edith Cowan University) | Capacity change |
|  | Longitudinal approach | Conceptualising CBA as occurring over a time period that can accommodate ‘good and bad days’. | *picture of progress along with the measurement over time.* (Faculty, Edith Cowan University) | Activities |
|  | Multiple perspectives & moments | Assessment comprises multiple perspectives on student performance that are collected at variety of moments in their performance and development. | *So rather than it being…all eggs in one basket …there's quite a range of different types…having a range of different ways of showing your competency.* (Graduate, Edith Cowan University) | Activities |
|  | Responsibilities & relationships | Responsibilities, duties, communication, and roles are reorientated and changed (with acceptance) which impacts on the learning and experience. This can be related to streamlining or processes to influence workload; low and high-stakes decisions; improved honest conversations. This includes reimagined relationships between users. | *it's more of a collaboration where you go, what we've seen eight weeks is taken into account, but it's also an assessment over multiple tasks and multiple settings. So yeah, I just feel like that's taking pressure off our educators. Because we do look back to Monash to monitor … they that have that type of role.* (Faculty, Monash University) | Reach & Reaction  ± Capacity change  ± Behaviour change |
|  | Shared belief | Stakeholders have a shared value and belief in PA – a team approach. | *the forms were very time consuming…but I never looked past the importance of getting them.* (Graduates, Edith Cowan University)  *we do very much feel like a team…we're facilitating the learning and the students taking ownership for that.* (Faculty, University of Canberra) | Capacity change |
|  | Student-led | Students are the leaders in their own learning and development. | *having the students come in and have an understanding…and taking responsibility for their own learning has been really valuable…previously a lot of the time ]was] spent around…me trying to identify a lot of those things. And so having the students know what they're looking for, is really valuable.* (Supervisors, University of Wollongong) | Reach & Reaction  Capacity change  Behaviour change |
| Hinderances | Accreditation & institutional requirements | Recognition of institutional and accreditation processes, policies and requirements that can hinder. Although this code is under hinderances, there may be helpful aspects. | *we've got a whole lot of university supports behind us as well, because the university's got amazing programmes.* (Faculty, Monash University) | Assumptions  Capacity change |
|  | Change | This incorporates all aspects of change which people find challenging i.e., taking time to accept, changing views and beliefs, resistance to new ideas. Also includes the non-challenging aspects of change i.e., it just takes time, and that it can be refreshing. | *…when we first implemented and there was a little bit of uncertainty…but then I think, after the implementation…we had a lot more clarity about how it's being markers, and it has been discussed and how the final decision was going to be reached. Once…people at the sites knew…the process itself was really robust. I think that that's when they felt more comfortable with it as well, because I think with any change, people do have lots of questions.* (Faculty, Edith Cowan University) | Capacity change |
|  | Extra effort & time | Acknowledging that more is required of users in terms of time and considered through (no longer ‘tick-and-flick’). | *it required more input and more insight. And I think that that has been a switch for me, like, oh, I actually have to be quite invested in this assessment for the student.* (Faculty, Edith Cowan University) | Capacity change |
|  | Limited user involvement | Intentional or unintentional restrictions placed on some users that inhibit their understanding of programmatic assessment which may, or may not, hinder their ability to meaningfully contribute to the system. | *They still struggled through, and then it didn't make much progress and don't know what happened at the end if they got through or not.* (Supervisor, Monash University) | Capacity change |
|  | Over-, forced, or mis-aligned assessment | Overassessment or assessment that is not authentic or does not align with the learning outcomes or professional standards. | *Sometimes…the forms were being done just because it was a particular point in time and they didn't always quite marry up with where you might have been in that project, because the projects were so varied.* (Graduate, Edith Cowan University) | Activities  Reach & Reaction |
|  | Stakeholder communication & coherence | Poor or insufficient process to enable communication and coherence between stakeholders. | *I could be more forceful and I could…just go away and say, “I've redesigned the assessment tasks, what do you think?”…but…my personality is to not like, piss people off…I would prefer to work collegially with them, I feel like I'll get my time to make the changes. And so maybe I just sit it out and try and understand the system a bit more…there are some big personalities.* (Faculty, Monash University) | Capacity change |
|  | User capability | Users need to understand the system and achieving this level of knowledge can be challenging with a range of barriers. This includes student and supervisor attributes, or lack thereof. | *there is variability in the students, and particularly if I'm with some of the international students ranging in countries, they're quite reticent to…participate…in the conversations…around…assessing competence, because you want it to be a shared thing…some students are more happy to, to kind of participate in that process than others. It's not, it's not a deal breaker, but yeah, it does…affect it.* (Supervisors, Edith Cowan University) | Capacity change |
|  | Work demands | Extraneous expectations, requirements and demands placed on users that can impact on their ability to undertake programmatic assessment. | *get increasing numbers…but no additional resourcing that's like the catch 22.* (Faculty, University of Wollongong) | Capacity change |
| Threats to sustainability | Onerous | Time and effort required from users. | *I think we did [over-assess] at the start, because we did have so many forms. Some portfolios were so big, it was overwhelming.* (Faculty, Edith Cowan University) | Capacity change |
|  | Philosophical dissonance | Difference in believes, values, and worldview between stakeholders. | *…one of the barriers could be sites not wanting to not embracing the student led aspect of it and wanting to have…that power.* (Faculty, Edith Cowan University) | Capacity change |
| Enablers to sustainability | Continuous evaluation & improvement | Embedded and continuous evaluation to streamline and improve the process for all users. | *getting the graduates inputs really valuable…that's probably really important element.* (Faculty, Monash University) | Capacity change  Activities |
|  | Equipping users & shared ownership | Ensuring all users have the required knowledge and skills. | *further education for the preceptors…that would probably be the main thing that would help sustainability.* (Faculty, Edith Cowan University) | Activities  Reach & Reaction |
|  | Normalisation | Becomes the new ‘norm’ or status quo for the university and the profession more broadly = culture change. This new ‘norm’ is manageable and accepted by stakeholders. NB: this may link into philosophical shift but is more of a speculation by participants. | *as we use it, more and more with the different cohorts…it becomes an established process. And I think once it's established that contributes to the sustainability of it as a model.* (Faculty, Edith Cowan University) | Direct benefit |
| Challenging situations | Conversations | Descriptions as to how discussions and discourse are used to navigate challenging situations. | *…it makes the hard conversations easier. I think it's more evident when it's spelt out.* (Supervisors, Monash University) | Behaviour change |
|  | Different opinions | Descriptions of situations where opinions were not in agreement and how these were navigated. | *there was some disagreement about one particular student, and so there was four staff and…some felt that she had achieved competency and others weren't sure…it was resolved in that the student…asked to provide further explanation and additional reflective piece to address the concerns…in that instance, we utilised really the fundamentals of programmatic assessment and explain the concern to the student and put the onus back on the student to respond to those concerns. And they did that successfully. And all the staff were happy with the outcome and the students subsequently progressed.* (Faculty, Edith Cowan University) | Behaviour change |
|  | Illuminating circumstances | Descriptions of how programmatic assessment allows for external or the broader context to be revealed and then integrated into assessment decisions. | *We had an incident this year…they hid it quite well from me until the final assessment…Monash are really, really good in every way for handling that.* (Supervisors, Monash University) | Behaviour change |
| Other |  |  |  |  |

ToC theory of change.
