# Supplementary material for: An evaluation of programmatic assessment across health professions education using contribution analysis
Source: Adv Health Sci Educ Theory Pract. 2025 Jun 4;31(1):211–38. doi: 10.1007/s10459-025-10444-5 (PMC12929344; doi:10.1007/s10459-025-10444-5)
Supplement: Supplementary file 6 — Supplementary Material 6 [file 10459_2025_10444_MOESM6_ESM.docx]

**Online Resource 6.** The initial (step 4) and revised (step 5, in shaded rows) contribution claims developed for programmatic assessment as part of contribution analysis and assessed using the Relevant Explanation Finder (Biggs et al., 2014; Delahais & Toulemonde, 2012; Lemire et al., 2012).

| **Contribution claim** | | **Classification ^a^** | **Influence and action ^b^** | **Degree of influence ^c^** | **Evidence-informed ^d^** |
| --- | --- | --- | --- | --- | --- |
| **No.** | **Label** |  |  |  |  |
| 1 | Leadership drove the transition to programmatic assessment | Non-identified | Confirmed | Medium | Moderate |
|  | Leaders drove transition | Non-identified | Confirmed | High | High |
| 2 | Training and experience built user capability | Co-mingled | Confirmed | Moderate | Moderate |
|  | Training developed capabilities of the people | Co-mingled | Confirmed | High | High |
| 3 | Multiple data-points, collected over time, are needed for high-stakes assessment decisions | Primary | Confirmed | High | High |
|  | High-quality low-stakes data-points underpinned high-stakes progression decisions | Primary | Confirmed | High | High |
| 4 | A learning team was fostered | Primary | Confirmed | High | High |
|  | A learning team was fostered | Primary | Confirmed | High | High |
| 5 | Progression moments enabled early detection of issues and remediation | Primary | Confirmed | High | High |
|  | Progression moments enabled early detection of issues and psychologically safe, learner-led remedial action | Primary | Confirmed | High | High |
| 6 | Lifelong learners became confident and prepared graduates | Primary | Confirmed | Moderate | High |
|  | Learners became prepared, safe, and confident graduates | Primary | Confirmed | Moderate | High |
| 7 | Programmatic assessment became the status quo | Non-identified | Confirmed | Moderate | Moderate |
|  | Programmatic assessment became the status quo | Non-identified | Confirmed | Moderate | High |
| 8 | Assessment was authentic and contextually responsive | Non-identified | Confirmed | Moderate | Moderate |
|  | Amalgamated into contribution claim 1 | | | | |
| 9 | Improved institutional reputation | Primary | Confirmed | Low | Low |
|  | Discarded due to insufficient evidence | | | | |

^a^ Classification was the categorisation in relation to how evidence explained causal link(s): primary (causal link identified in the ToC accounts for and explains observed outcomes), non-identified (causal link, different from that given in ToC accounts for and explains observed outcomes), co-mingled (non-identified and primary causal link both contribute to and explain observed outcomes).

^b^ Influence and action is the determination if evidence confirmed, refuted, or contributed to the causal link(s).

^c^ Overall degree of influence included certainty (degree which observed outcome matched that predicted in theory of change), robustness (degree which causal link(s) was a significant contributor), range (degree which causal link(s) contributed to range of observed outcomes), prevalence (degree which causal link(s) contributed to observe outcomes across settings), applied using the matrix reported by Biggs et al. (2014) with the ratings of levels of low, moderate, and high used.

^d^ Evidence-informed is the credibility and rigour of verifying evidence, applied using the matrix reported by (Biggs et al. 2014) with the ratings of low, moderate, and high used.
