# Supplementary material for: An evaluation of programmatic assessment across health professions education using contribution analysis
Source: Adv Health Sci Educ Theory Pract. 2025 Jun 4;31(1):211–38. doi: 10.1007/s10459-025-10444-5 (PMC12929344; doi:10.1007/s10459-025-10444-5)
Supplement: Supplementary file 7 — Supplementary Material 7 [file 10459_2025_10444_MOESM7_ESM.docx]

**Online Resource 7.** Summary of literature review conducted in step 5 of contribution analysis for programmatic assessment with mapping to the theory of change.

| **Authors and year** | **Discipline(s)** | **Research aim** | **Participants**  **(type, number)** | **Mapping to the Theory of Change** |
| --- | --- | --- | --- | --- |
| Baartman et al. (2022) | Health, paramedicine, teaching, communication | Explore design choices made in programmatic assessment. | Educators (n = 9) | Confirmed and elaborated contribution claims 1, 3, 4, 5, 7. |
| Baartman et al. (2023) | Communication sciences | Explore low and high performing students’ experiences related to feedback-seeking behaviour within programmatic assessment. | Students (n = 16, 55) | Confirmed contribution claims 2, 4, 5. |
| Dart et al. (2021) | Dietetics | Evaluate the implementation of programmatic assessment and explore its ability to support students and assessors. | Graduates (n = 6)  preceptors (n = 12)  faculty (n = 9) | Confirmed contribution claims 1, 2, 3, 4, 5, and threat 2. |
| de Jong et al. (2022) | Veterinary | Explore the influence of narrative information in perceiving saturation of information during high-stakes decision-making. | Competency committee members (n = 7) | Confirmed and elaborated contribution claims 3 and 4. |
| Jamieson et al. (2022) | Dietetics | Explore how supervisors experienced and perceived their positioning within programmatic assessment. | Preceptors (n = 44) | Confirmed threat 3. |
| Roberts et al. (2022) | Medicine | Unpack underlying causes that explain student experience of programmatic assessment. | Learners (n = 112) | Confirmed and elaborated contribution claims 1, 2, 3, 4, 5, external influence 1, and threat 2. |
| Ross et al. (2023) | Medicine | Evaluate the implementation of competency-based medical education with an emphasis on programmatic assessment. | Publications (n = 13) | Confirmed and elaborated contribution claims 1, 4, 5, 6, 7. |
| Schut et al. (2020) | Medicine | Explore how teachers conceptualise assessment within programmatic assessment and how they engage with learners in assessment relationships. | Teachers (n = 23) | Confirmed and elaborated contribution claims 3, 4, and threat 2 and 3. |
| Schut et al. (2021) | Health  professions | Examine current research on programmatic assessment. | Publications (n = 27) | Confirmed and elaborated contribution claims 1, 2, 3, 4, 5 and threat 1 and 2. |
| Torre et al. (2022) | Medicine, dentistry, veterinary | Understand factors affecting implementation process of programmatic assessment how specific implementation challenges are managed. | Educators (n = 6) | Confirmed and elaborated contribution claims 1, 2 and external influence 2. |
